# Systematic Development and Characterization of Enzyme-Free, Borax-Crosslinked Microneedles for Glucose-Responsive Insulin Delivery and In Vivo Glycemic Mitigation

**DOI:** 10.3390/pharmaceutics17121578

**Published:** 2025-12-08

**Authors:** Cuc Thi Dinh, Linh Phuong Nguyen, Uyen Thu Pham, Anh Mai Nguyen, Hanh Thi My Do, Toan Quoc Tran, Phuong Duc Luu, Tien Duy Doan, Mo Thi Hong Bui, Duong Thanh Nguyen

**Affiliations:** 1 Institute of Chemistry, Vietnam Academy of Science and Technology, 18 Hoang Quoc Viet St., Nghia Do Ward, Hanoi 10000, Vietnam; dtcucvhh@gmail.com (C.T.D.); tranquoctoan2010@gmail.com (T.Q.T.); luuphuongtheday@gmail.com (P.D.L.); doanduytien@yahoo.com (T.D.D.); ledao.9996@gmail.com (M.T.H.B.); 2 School of Population Health, University of New South Wales, Sydney, NSW 2052, Australia; nplinh239@gmail.com; 3 Institute of Life and Environmental Sciences, 38/10 Ho Dac Di St., Dong Da Ward, Hanoi 10000, Vietnam; phamuyenpjj@gmail.com (U.T.P.); manhng293@gmail.com (A.M.N.); myhanh141001@gmail.com (H.T.M.D.); 4 Faculty of Chemistry, Graduate University of Science and Technology, Vietnam Academy of Science and Technology, 18 Hoang Quoc Viet St., Nghia Do Ward, Hanoi 10000, Vietnam; 5 Institute of Materials Science, Vietnam Academy of Science and Technology, 18 Hoang Quoc Viet St., Nghia Do Ward, Hanoi 10000, Vietnam

**Keywords:** glucose-responsive microneedles, smart drug delivery, insulin delivery, diabetes mellitus, enzyme-free, borate–diol chemistry, PVA/Dextran hydrogel, glycemic stability, hypoglycaemia, transdermal drug delivery

## Abstract

**Background:** Conventional insulin injections cannot mimic physiological pancreatic function and often lead to dangerous hypoglycemic events that glucose-responsive systems aim to prevent. Glucose-responsive microneedles (MNs) offer a promising closed-loop alternative. We developed an enzyme-free, glucose-responsive MN patch composed of a PVA/Dextran hydrogel dynamically crosslinked with borax, and evaluated its performance, biosafety, and in vivo efficacy. **Methods:** MNs were fabricated from PVA/Dextran via micromolding and crosslinked with borax. The formulation was systematically optimized based on mechanical properties and glucose-responsive release kinetics. Physicochemical properties, biosafety (cytotoxicity, skin barrier recovery, boron leaching), and in vivo efficacy in a type 1 diabetic mouse model were evaluated in comparison to a subcutaneous (SC) insulin injection. **Results:** The optimized MNs showed robust mechanics (per-needle fracture force approximately 1.0 N) for reliable skin penetration. The system demonstrated clear glucose sensitivity, with a release flux ratio ≥1.5 between hyperglycemic (e.g., 400 mg·dL^−1^) and normoglycemic (100 mg·dL^−1^) conditions and exhibited excellent reversibility under alternating glucose levels. The patch was highly biocompatible, with >95% cell viability, the only transient skin barrier disruption that fully recovered within 24 h, and had low boron release from patches in vitro. In vivo, the optimized sI-MN patch demonstrated a sustained, glucose-responsive release profile, maintaining blood glucose in diabetic mice near 100 mg·dL^−1^ for approximately 8 h. This pharmacokinetic profile contrasts markedly with the rapid hypoglycemic nadir and rebound hyperglycemia observed with a standard subcutaneous insulin bolus, highlighting the patch’s potential for mitigating hypoglycemia. **Conclusions:** The enzyme-free PVA/Dextran/borax MN patch enables autonomous, glucose-responsive insulin delivery. It provides more stable and safer glycemic control than conventional injections by mitigating the risk of hypoglycemia. By mitigating the hypoglycemic risk associated with bolus injections, this systematically optimized platform represents a potential step toward a safer, patient-friendly diabetes therapy, though significant challenges in duration and dose scaling remain.

## 1. Introduction

Diabetes mellitus represents a rapidly escalating global health crisis, with projections indicating that the number of affected adults will surge from approximately 589 million to 853 million by 2050 [1]. For patients with type 1 diabetes and many with advanced type 2 diabetes, insulin therapy remains the cornerstone of management [2]. However, the standard regimen of multiple daily subcutaneous (SC) injections is inherently painful, inconvenient, and places a significant burden on patient adherence and quality of life [2,3]. More critically, manual dosing via SC injection cannot effectively mimic the physiological, pulsatile insulin secretion of a healthy pancreas, frequently leading to dangerous fluctuations in blood glucose levels [2,3]. The resulting glycemic variability often precipitates both chronic hyperglycemic complications and, most acutely, severe hypoglycemia—a life-threatening condition that remains the primary barrier to safe and effective insulin intensification [2,4]. This clinical reality underscores a sustained and urgent demand for safer, more patient-friendly, and physiologically responsive insulin delivery strategies.

A paradigm shift toward mimicking pancreatic function is offered by glucose-responsive (GR) insulin delivery systems, often referred to as “smart insulin” [5]. These closed-loop platforms are designed to autonomously adjust insulin release in real time based on ambient glucose concentrations, thereby mitigating the severe glycemic swings associated with manual dosing [5,6,7]. Clinical evidence strongly supports this approach, demonstrating that closed-loop systems significantly improve the time spent in the target glucose range while substantially reducing the risk of hypoglycemia [7]. Early and widely explored GR strategies have primarily relied on enzyme-based mechanisms, such as those utilizing glucose oxidase (GOx) [8,9,10]. While effective for glucose sensing, GOx-based systems generate hydrogen peroxide H_2_O_2_—a cytotoxic byproduct—and suffer from inherent limitations, including enzyme denaturation, loss of activity, and sensitivity to pH changes, which severely restrict their long-term durability and clinical translatability [4,8,9,10].

To overcome the instability and biosafety concerns of enzyme-based systems, research has shifted toward developing robust, enzyme-free alternatives. One prominent approach involves the use of phenylboronic acid (PBA) derivatives, which can form reversible covalent bonds with diol-containing molecules like glucose [11,12,13]. However, the clinical translation of PBA-based systems is often hindered by the need for complex chemical modifications to achieve glucose sensitivity at physiological pH [12,14], as well as potential concerns regarding the cytotoxicity of boronic acid derivatives at high concentrations [14,15]. Therefore, a critical knowledge gap exists for a simple, highly biocompatible, and stable enzyme-free GR system that can be easily scaled for clinical application.

Meanwhile, microneedle (MN) arrays have emerged as a transformative and patient-friendly platform for transdermal drug delivery [5,16,17]. These micron-scale structures painlessly breach the outermost skin barrier, the stratum corneum, to create transient microchannels that dramatically enhance drug permeability while remaining minimally invasive. Among various formats, dissolving microneedles (DMNs) are particularly promising [3,7,9,10,18,19]. Fabricated from biocompatible and biodegradable polymers, DMNs completely dissolve in the skin after application, releasing their payload without generating sharp medical waste [6,20]. Poly(vinyl alcohol) (PVA) is an ideal polymer for DMNs due to its excellent water-solubility, biocompatibility, and robust film-forming capacity, allowing for the fabrication of needles with reproducible geometry and high skin-penetration efficiency [3,10,21,22,23].

To address the aforementioned limitations of existing GR systems, we report the development of a novel, enzyme-free, glucose-responsive insulin delivery system integrated into a dissolving PVA-based microneedle patch [12,24]. Our system leverages the simple and highly biocompatible borate–diol chemistry through the dynamic crosslinking of PVA and Dextran with borax [12,24]. This approach exploits the reversible borate–diol ester bonding, which is sensitive to glucose via a pH-independent competitive binding mechanism [12,24]. The strategic incorporation of Dextran, a polysaccharide rich in cis-diol groups, serves to amplify this glucose-responsive behavior by increasing the crosslink density and hydrogel swelling capacity, thereby promoting rapid, on-demand insulin release during hyperglycemic events [10,14,24].

**Figure 1:**
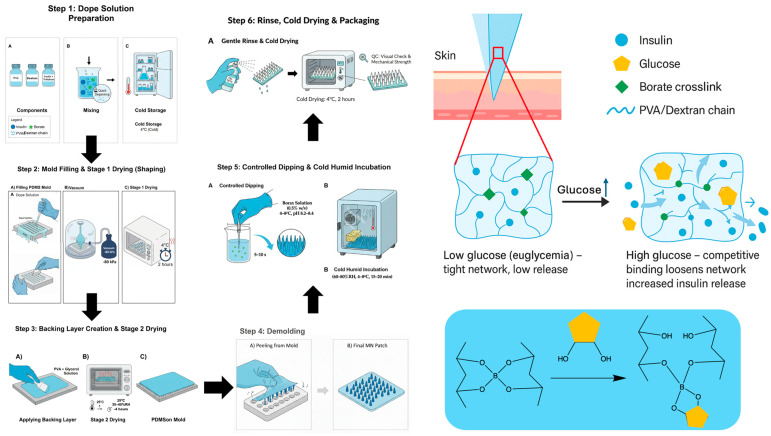
Schematic of insulin-loaded PVA/dextran microneedle (sI-MN) fabrication with controlled post-dip borate activation. (Step 1) Polymer dope. A cold, sterile blend of PVA and dextran is prepared to total solids of 18–22% (*w*/*w*) with trehalose (2% *w*/*v*) and recombinant human insulin (5 mg·mL^−1^). (Step 2) Mold filling. The dope is dispensed onto PDMS-negative molds and driven into the cavities under vacuum (−60 to −90 kPa; 2–3 cycles × 60–120 s) until fully filled and bubble-free; the array geometry is 14 × 14 (about 150 functional needles within the active area), with individual needles of 900 µm height and 400 µm base diameter. Molds are then cold-dried at 4 °C for 2 h. (Step 3) Backing layer application and Stage-2 drying. A backing layer (PVA 15% *w*/*w* + glycerol 0.5% *w*/*w*) is cast to cover the mold and dried at 25 °C for 3–4 h (RH 35–40%) to mass-constant. (Step 4) Demolding and trimming. Patches are released and cut to size (e.g., Ø 15 mm for product patches; scaled as needed for mouse dosing). (Step 5) Controlled borax post-dip—full-array immersion (single pass). The entire dry microneedle array is immersed once in chilled sodium tetraborate solution (0.5% *w*/*v*, pH 8.2–8.4, 4–8 °C) for 5–10 s to form dynamic borate crosslinks throughout the array, then incubated at 4–8 °C, 60–80% RH for 15–20 min. (Step 6) Cold, humid incubation and finishing. Gentle PBS mist-rinse (3–5 s), cold-drying at 4 °C for 2 h, QC check, and sealing with desiccant for storage at 2–8 °C. Mechanism: at low glucose, borate crosslinks stabilize the PVA/dextran network, retaining insulin; at high glucose, competitive glucose–borate binding loosens the network to increase insulin diffusion (reversible, enzyme-free). Symbol key: insulin (blue), glucose (yellow), borate crosslinks (green), and PVA/Dextran chain (blue wavy line).

While borate–diol chemistry is an established mechanism for glucose-responsive materials, its translation into robust transdermal devices has been hampered by difficulties in achieving sufficient mechanical strength for reliable skin penetration while simultaneously preserving tunable, rapid glucose-responsive release kinetics [25]. We hypothesize that these competing requirements can be met through a systematic, multi-step optimization process. By sequentially gating formulation variables (polymer ratio, total solids, insulin load, and crosslinking time) against a priori performance criteria (e.g., fracture strength ≥ 1.0 N, flux ratio R_0–6h_ ≥ 1.5×), we can identify a PVA/Dextran/borax formulation that is both mechanically robust and highly responsive [26,27]. Herein, we present the comprehensive in vitro and in vivo evaluation of this optimized microneedle patch, demonstrating its (i) sufficient mechanical strength for reliable skin penetration; (ii) rapid skin barrier recovery and excellent biosafety; (iii) tunable, enzyme-free glucose-responsive insulin-release kinetics in vitro; and (iv) superior efficacy in maintaining stable and safe glycemic control in vivo in a type 1 diabetic mouse model, critically mitigating the risk of the hypoglycemic nadir common with SC injections. This simple and scalable platform represents a significant step toward safer, patient-friendly, and autonomous diabetes therapy. An overview of the device concept and fabrication workflow is shown in Figure 1.

## 2. Materials and Methods

### 2.1. Materials

Poly(vinyl alcohol) (PVA, 98–99% hydrolyzed, Mw 85–124 kDa) and dextran (40–70 kDa) were obtained from Sigma-Aldrich (St. Louis, MO, USA). Sodium tetraborate decahydrate (borax, ≥99.5%) and trehalose dihydrate (≥99%) were from Sigma- Aldrich as well. Zinc-stabilized (research grade) recombinant human insulin was purchased from Sigma- Aldrich and stored at 2–8 °C per the manufacturer’s instructions. Phosphate-buffered saline (PBS, pH 7.4), Dulbecco’s PBS, DMEM high glucose, fetal bovine serum (FBS), and penicillin–streptomycin were from Thermo Fisher Scientific (Waltham, MA, USA). Glycerol (USP/Ph.Eur.) was from Merck KGaA (Darmstadt, Germany). D-glucose (cell culture and analytical grade), and Eosin Y were from Sigma- Aldrich (St. Louis, MO, USA).

Polydimethylsiloxane (PDMS) base and curing agent (Sylgard™ 184) were obtained from Dow (Midland, MI, USA) and used to cast negative molds. Parafilm M^®^ was from Bemis Company (Neenah, WI, USA). Semi-occlusive medical adhesive film (Tegaderm™, 3M Company, Saint Paul, MN, USA) used for patch fixation was from 3M (St. Paul, MN, USA). Sterile syringe filters (0.22 µm, PVDF or PES) and low-protein-binding microcentrifuge tubes were from MilliporeSigma (Burlington, MA, USA). Black, clear-bottom 96-well plates for spectrophotometric assays were from Corning (Corning, NY, USA).

For plasma insulin quantification, a human insulin ELISA (Mercodia AB, Uppsala, Sweden) was used. Calibrators were prepared in ng·mL^−1^ and fitted with a 4-parameter logistic (4PL, 1/y^2^). The validated analytical range used for this study was 0.30–10 ng·mL^−1^ (LLOQ 0.30 ng·mL^−1^; estimated LoD 0.10 ng·mL^−1^). All plates included duplicate standards/QCs; runs required R^2^ ≥ 0.99, QC recovery 80–120% (≤20% at LLOQ). For confirming negligible endogenous secretion in STZ mice when required, a mouse C-peptide ELISA (Crystal Chem, Elk Grove Village, IL, USA; calibrator range 0–10 ng·mL^−1^) was employed. Dextrose 50% (*w*/*v*) solution for IVGTT was from B. Braun (Melsungen, Germany). Isoflurane for brief anesthesia during patch application and blood sampling was from Baxter (Deerfield, IL, USA).

Boron leaching was quantified using TraceCERT^®^ boron ICP-MS standards (Sigma-Aldrich) prepared in ultrapure nitric acid for trace analysis (Merck KGaA). Working calibration ranges of 0–100 µg·L^−1^ were freshly prepared in acidified matrix as specified in the corresponding method section. All solutions contacting insulin were prepared with endotoxin-free water, filtered through 0.22 µm membranes, and handled aseptically in a Class II biosafety cabinet. Unless otherwise stated, reagents were of anal ytical grade or higher and used as received.

### 2.2. Methods

#### 2.2.1. Fabrication of PVA/Dextran/Borax/Insulin Microneedle Arrays

##### Mold Design and Replication

Microneedle (MN) array layouts (square matrix, conical frustums; nominal base 400 µm; height 900 µm) were drafted in vector software and laser-engraved into PMMA plates to generate negative masters. Engraving was performed using a laser system (manufactured in Guangzhou, China; Phan Long Laser–monitored in Hanoi, Vietnam) operated at 20% laser intensity and 600 mm·s^−1^ for the selected pattern settings (speed range explored: 500–1400 mm·s^−1^; power range explored: 10–100%). PDMS working molds (Sylgard™ 184, 10:1 base/curing agent, *w*/*w*) were prepared by mixing, vacuum degassing for 30 min, casting onto the PMMA master, and curing at room temperature for 24 h. After curing, molds were peeled, inspected for defects, cleaned with 70% ethanol, and stored dust-free prior to replica molding [28]. Mold filling used vacuum cycles (−60 to −90 kPa, 2–3 cycles × 60–120 s) to ensure void-free cavities. Prior to use, PDMS molds were cleaned (70% ethanol), air-dried in a clean hood, and UV-irradiated to ensure surface cleanliness. Where reverse replication was required, a thin metallic (Ag) sputter was briefly applied to the PMMA surface to improve feature fidelity; production runs used untreated PDMS molds.

##### Microneedle Fabrication Workflow (Schematic in Figure 1)

In this study, PDB-MN denotes poly(vinyl alcohol)/Dextran/borax microneedle patches; sI-MN refers to insulin-loaded PDB-MN.

Step 1—Dope preparation (no borax): a cold aqueous PVA/dextran blend was prepared at 18–22% *w*/*w* total solids with PVA/Dextran = 90:10 (*w*/*w*) and trehalose 2% *w*/*v*; recombinant human insulin (5 mg·mL^−1^) was gently incorporated under cold conditions with minimal aeration. No borax was included in the dope to avoid premature gelation and to preserve tip fidelity.

Step 2—Mold filling and Stage-1 cold-drying: 70 µL per 15 mm disk of dope was dispensed onto PDMS-negative molds (working array 14 × 14; nominal needle height 900 µm, base 400 µm) and driven into the cavities by vacuum −60 to −90 kPa (2–3 cycles × 60–120 s) until void-free; the surface was then leveled with a doctor blade. Immediately after filling, molds were placed in a closed chamber at 4 °C for 2 h to stabilize needle shape while maintaining insulin integrity.

Step 3—Backing layer and Stage-2 drying: a backing layer (PVA 15% *w*/*w* + glycerol 0.5% *w*/*w*) was cast to cover the mold and arrays were dried to completion at 25 °C for 3–4 h (RH 35–40%) in a closed desiccator to mass-constant (±1 mg per patch over 30 min).

Step 4—Demolding and trimming: arrays were released from the molds and trimmed to the required format (e.g., 15 mm disks or mouse-scaled mini-patches), avoiding deformation of the needle field.

Step 5—post-dip borax and cold–humid incubation: Entire patches (needles + backing) were immersed once in chilled sodium tetraborate 0.5% *w*/*v* (pH 8.2–8.4, 4–8 °C) for 5–10 s, then incubated at 4–8 °C and 60–80% RH for 15–20 min to complete reversible boronate–diol crosslinking.

Step 6—Finishing and storage: a fine PBS mist (3–5 s) was applied to normalize surface ionic strength and remove loosely bound borate, followed by cold-drying at 4 °C for 2 h. Patches were sealed with desiccant in foil pouches and stored at 2–8 °C; prior to in vivo use, they were equilibrated sealed at room temperature for 15–20 min and then opened and applied. All steps were conducted at ≤30 °C under clean, low-airflow conditions.

##### Design of Optimization Experiments

To identify a single, manufacturable recipe that meets all pre-specified performance gates, we executed a four-step, sequential optimization with explicit factor levels, standardized readouts, and decision rules. Unless otherwise stated, each condition was produced in ≥3 independent batches (“patch-level” replicates), mechanical testing sampled ≥10 needles per patch, and release assays were run in technical triplicate per batch. For single-needle testing, needles were selected using stratified sampling across the array (center and peripheral zones) to capture potential spatial heterogeneity from casting/drying. This sampling density was chosen as a practical compromise between throughput and representativeness and is supported by the low defect rate (<5%) and high mechanical reproducibility observed across independent arrays. Test order was randomized where feasible and data processing was blinded to factor levels. Primary acceptance thresholds were defined a priori (Table 1): mean fracture strength ≥ 1.0 N·needle^−1^ (borderline acceptable ≥ 0.8 N·needle^−1^), glucose-responsiveness ratio R_0–6h_ ≥ 1.5×, encapsulation efficiency (EE) ≥ 70–80%, insulin loss during borax dip ≤ 5% of nominal load, and boron leaching < 10 µg·patch^−1^·24 h^−1^. Glucose-responsiveness was computed from cumulative insulin-release profiles in PBS (pH 7.4, 37 °C) containing either 100 or 400 mg·dL^−1^ D-glucose: a linear slope (flux, µg·h^−1^) was fit over 0–6 h for each glucose level, and R_0–6h_ was defined as flux(400)/flux(100).

Step 1—Select PVA/dextran ratio at fixed solids and post-dip configuration. We screened PVA/dextran mass ratios of 95:5, 90:10, 85:15, 80:20, and 75:25 (*w*/*w*) at a fixed total solids of 20% (*w*/*w*). To avoid confounding composition with crosslinking intensity, all candidates received the same post-dip activation: a single brief immersion in chilled, mildly alkaline borax (0.5% *w*/*v* sodium tetraborate, pH 8.2–8.4, 4–8 °C). Primary readouts were (i) per-needle fracture strength measured on a texture analyzer using a flat platen and controlled approach speed until catastrophic failure (10 needles per patch; ≥3 patches per ratio), and (ii) glucose-responsiveness R_0–6h_ derived from ELISA-based quantification of insulin release at 100 vs. 400 mg·dL^−1^ glucose. A supportive readout—swelling at 6 h (%) in 0/100/400 mg·dL^−1^—was recorded to guard-band mechanical integrity. The decision rule was to select the lowest-variance ratio whose patch-level mean fracture was ≥1.0 N·needle^−1^ and R_0–6h_ was ≥1.5×; ties were broken by larger R_0–6h_ and lower swelling at 0 mg·dL^−1^.

Step 2—Select total solids at the Step-1 ratio. With the Step-1 ratio fixed, we varied total solids across 18, 19, 20, 21, and 22% (*w*/*w*), keeping the borax bath and immersion identical to Step-1. The same readouts (fracture and R_0–6h_) were collected, with optional 6 h swelling as a manufacturability check (molding/demolding defects, tip fidelity). The decision rule was to select the lowest-variance solids level that maintained fracture ≥ 1.0 N·needle^−1^ and maximized R_0–6h_, provided arrays molded and released cleanly from the PDMS without defects.

Step 3—Select insulin concentration in the dope at the chosen ratio/solids. Nominal insulin concentrations of 2.5, 5.0, 7.5, and 10.0 mg·mL^−1^ were prepared under cold, sterile handling. Entire patches were solvent-extracted in PBS (pH 7.4, 37 °C) with gentle agitation until complete dissolution; clarified extracts (centrifugation/0.22 µm) were quantified by sandwich ELISA against a multi-point insulin calibration. Readouts were encapsulation efficiency (EE, %) and drug loading (IU·patch^−1^). The decision rule was to select the concentration that achieved the target IU·patch^−1^ with EE within the acceptance band (Table 1) and an acceptable between-patch coefficient of variation; ties were resolved by higher EE and lower CV.

Step 4—Confirm borax immersion time at fixed composition. At the Step-1/2/3 composition, we evaluated post-dip immersion times of 5, 7, 10, 12, and 15 s in the same chilled borax bath. Primary readouts were (i) insulin loss to the bath (%) measured by ELISA after immediate neutralization and ≥1:20 dilution into ELISA sample diluent; spike-recovery in borax matrix confirmed 80–120%, and (ii) boron leaching after 24 h incubation in PBS at 37 °C (µg·patch^−1^·24 h^−1^), quantified by ICP-MS with calibration/QA checks. A brief guard-check of R_0–6h_ verified that responsiveness was preserved. The decision rule was to adopt the shortest immersion time that simultaneously kept insulin loss ≤ 5% and boron leaching < 10 µg·patch^−1^·day^−1^ while maintaining R_0–6h_ ≥ 1.5×; conditions breaching the boron limit (e.g., 15 s) were pre-specified for exclusion.

Applying this gated, sequential design converged on a single composition/processing recipe that satisfied all acceptance thresholds with favorable variance. It is crucial to differentiate this “gated, sequential” selection process from a formal Design of Experiments (DoE) optimization. This workflow was designed to efficiently identify a single compliant formulation that meets all a priori acceptance thresholds (Table 1), not necessarily to map the entire design space or find a global mathematical optimum. For example, the selection of 7 s in Step 4 was based on it being the minimum effective time that satisfied all safety and efficacy gates (R_0–6h_ ≥ 1.5×, Boron < 10 µg·patch^−1^), although 10 s also demonstrated compliance. This pragmatic approach balances performance with manufacturability and was deemed sufficient for this proof-of-concept validation.

#### 2.2.2. Optical Microscopy for Array Geometry and Morphology

Microneedle (MN) arrays were imaged using a calibrated optical microscope (stereo/bright-field). Prior to measurements, the imaging scale was verified with a stage micrometer at each magnification used. Single-needle height, base diameter, and tip radius were measured from micrographs in ImageJ 1.54 software(NIH). For each experimental condition, the number of needles and arrays analyzed is specified in the corresponding figure legends. Geometric defect rate (% malformed needles per patch) and effective needle count (needles conforming to design specifications per patch diameter) were calculated to describe manufacturing quality.

#### 2.2.3. Insulin Loading, Content Uniformity and Patch Stability

For patch content and content uniformity, entire patches were dissolved in PBS (pH 7.4, 37 °C) or the ELISA sample diluent recommended by the manufacturer, with gentle vortexing and brief sonication as needed to ensure complete dissolution. Solutions were clarified by low-speed centrifugation and, when required, by 0.22 µm filtration. Human insulin concentration was determined using a validated sandwich ELISA. Calibration curves (≥5 standards) were fitted with a four-parameter logistic (4PL, 1/y^2^) model with run acceptance R^2^ ≥ 0.99; calibrator/QC back-calculation within ±15% (±20% at LLOQ). Assay validation in patch-extract matrices and in mouse plasma is summarized in Supplementary Tables S1 and S2, respectively. Patch content (IU/patch) was obtained from ELISA-derived mass using the conversion 1 IU = 0.0347 mg human insulin before normalization.

For storage stability, patches were stored at 2–8 °C with/without desiccant and at room temperature (RT) with/without desiccant for predefined intervals. At each timepoint, patch insulin content was quantified by ELISA as described above and expressed relative to the initial value to track immunoreactive insulin retention over time.

To functionally confirm that immunoreactive insulin quantified in patch extracts remained bioactive after storage, a subset of freshly prepared and stored sI-MN patches was directly tested in STZ-diabetic mice following the in vivo protocols in Section 2.2.9.

Encapsulation efficiency (EE, %) was calculated as follows:

EE = (measured insulin content per patch/theoretical insulin mass dispensed into the mold) × 100

Theoretical insulin mass was computed from the dope insulin concentration and the dispensed dope volume per patch (70 µL per 15 mm disk). Drug loading is reported as the absolute insulin content (IU·patch^−1^), whereas EE captures process retention relative to the theoretical load (i.e., losses during molding/drying/post-dip).

#### 2.2.4. Mechanical Testing (Array-Scale and Single-Needle Fracture)

Array Force–Displacement. The bulk mechanical properties of the arrays were evaluated using a texture analyzer (TA.XTplus, Stable Micro Systems, Godalming, UK) with a 5 kg load cell. A 2 mm diameter cylindrical probe compressed the array at a constant rate of 0.5 mm·s^−1^. The resulting force–displacement data were normalized by the probe area to generate a stress–strain curve (N·cm^−2^), from which yield and fracture points were identified.

Single-Needle Fracture Force. The fracture force of individual needles was measured by micro-compression using the same texture analyzer setup. The array was fixed onto a rigid stage, and the probe was lowered at 0.5 mm·s^−1^ to compress a single needle until the point of fracture, defined as the peak force (N) recorded [29]. Sample sizes (needles per patch and patches per group) are indicated with the results. Mechanical outcomes are reported as mean ± SD; where applicable, reproducibility metrics (e.g., coefficient of variation or intraclass correlation) are provided.

#### 2.2.5. Insertion, Depth Profiling, and Local Barrier Function

Insertion (ex vivo, porcine skin). Arrays designed for insertion verification were fabricated with eosin Y incorporated in the needle tips. After application to excised porcine skin under standardized thumb pressure for a fixed dwell time, patches were removed and the skin surface was immediately examined by optical microscopy.

Transepidermal Water Loss (TEWL). Skin barrier disruption and recovery were quantified using an open-chamber tewameter (Tewameter^®^ TM 300, Courage + Khazaka electronic GmbH, Cologne, Germany). Measurements were taken at pre-, 0, 1, 2, 4, 6, 12, and 24 h post-removal; values were normalized to pre-application baseline. All TEWL measurements were conducted in a controlled room (20–25 °C; 40–60% RH). Animals were acclimatized for 15–30 min at rest with the dorsal site exposed immediately before each read. The acclimatization duration was selected in accordance with established guidance for TEWL measurements [33,34]. Three consecutive readings per site were recorded with the probe held perpendicular to skin; the median value was used for analysis. For each timepoint, n = 5 independent skin sites were measured (three technical readings per site averaged). The following groups were assessed: Baseline (no patch), blank PDB-MN, and insulin-loaded sI-MN.

#### 2.2.6. In Vitro Swelling and Glucose-Responsive Release

Swelling kinetics. Microneedle tips were incubated in PBS (pH 7.4, 37 °C) containing glucose at normoglycemic (100 mg·dL^−1^), hyperglycemic (400 mg·dL^−1^), or glucose-free (0 mg·dL^−1^) concentrations. At timepoints from 0 to 24 h, samples were weighed to determine water uptake. The percentage swelling was calculated as follows:

% Swelling = (W_t_ − W_0_)/W_0_ × 100

Cumulative release and on–off cycling. Insulin release was assessed in static/Franz diffusion setups maintained under sink conditions in PBS (pH 7.4) at 37 °C with glucose at 0, 100, or 400 mg·dL^−1^. Samples were collected over 0–24 h with medium replacement. For reversibility tests, donor media were alternated between 100 and 400 mg·dL^−1^ glucose at fixed intervals (30–60 min) for three cycles. Insulin concentrations in all receiver samples were determined by ELISA (4-PL fit; appropriate dilution into ELISA diluent; spike-recovery 80–120% verified in the receiver matrix). Initial release flux (0–6 h) was estimated from the slope of cumulative amount vs. time over the 0–6 h window, normalized by the effective release area. The flux ratio R_0–6h_ was defined as Flux(400 mg·dL^−1^)/Flux(100 mg·dL^−1^). The response time, T90, was defined as the time to reach 90% of the new steady-state release rate after a glucose switch.

#### 2.2.7. Boron Leaching

Patches were incubated 24 h at 37 °C in PBS; supernatant was analyzed by ICP-MS (Agilent 7700, Agilent Technologies, Inc., Santa Clara, CA, USA). Calibration employed TraceCERT^®^ boron standards (0–100 µg·L^−1^) in acidified matrix, with procedural blanks and matrix-matched QCs in each run; calibration R^2^ ≥ 0.99 and QC recovery 80–120% were required. Analytical method validation is provided in Supplementary Table S3. Boron release was reported as µg patch^−1^ day^−1^, with a predefined acceptance threshold of <10 µg patch^−1^ day^−1^.

#### 2.2.8. Animals, Diabetes Induction, Randomization and Blinding

Male C57BL/6J mice, 56–70 days old (8–10 weeks) and weighing 23–28 g at allocation, were housed under standard conditions with ad libitum access to food and water. Type 1 diabetes was induced by intraperitoneal streptozotocin (55 mg kg^−1^ daily for 5 days). Mice with fasting plasma glucose (FPG) > 300 mg·dL^−1^ were considered diabetic and enrolled. Individual induction outcomes are summarized in Supplementary Table S4. Animals were randomized to groups with allocation concealment; outcome assessors were blinded to treatment. All procedures were approved by the Institutional Animal Care and Use Committee (IACUC; protocol ILES-IACUC-2025-002, approved 11 July 2025) and complied with ARRIVE guidelines. Animals were housed at 22 ± 2 °C, 45–65% RH under a 12 h light/12 h dark cycle with environmental enrichment (nesting material), standard chow and water ad libitum. For blood sampling and patch application, brief isoflurane anesthesia was used (Materials). Humane endpoints: >20% body-weight loss from baseline, persistent FPG > 600 mg·dL^−1^ despite treatment, or any severe distress prompted euthanasia per IACUC policy.

#### 2.2.9. In Vivo Studies: Baseline Efficacy, GTTs, Feeding, and PK

Treatments and Dosing. Treatments included sI-MN mini-patches (Ø 3–5 mm, delivering 0.05–0.20 IU), blank PDB-MN patches (poly(vinyl alcohol)/Dextran/borax microneedle patches without insulin), a dose-matched SC insulin injection, and a PBS sham control. Patches were applied to a shaved dorsal skin site and secured with a semi-occlusive film. The nominal dose for mouse studies (0.05–0.20 IU) was delivered using “mini-patches” (Ø 3–5 mm) punched from larger, fully characterized patches (Ø 15 mm). The nominal dose was calculated based on this area ratio:

[Nominal IU per mini-patch] = (Mean IU per full patch) × (mini-patch area/full patch active area).

This calculation carries the critical, unverified assumption of perfect intra-patch homogeneity (i.e., that drug content is uniform across the entire patch surface, with no “edge effects” from drying or molding). As only inter-patch uniformity was validated (Figure S1), all subsequent pharmacokinetic and pharmacodynamic data based on this nominal dose must be interpreted with this limitation in mind.

Baseline Efficacy (0–12/24 h). Plasma glucose levels (PGLs) were monitored for 12–24 h using a glucometer. Plasma insulin was quantified by ELISA at selected timepoints. The area under the curve (AUC_0–12/24_) was calculated to assess overall glycemic control.

IVGTT. An intravenous glucose-tolerance test (IVGTT) was performed 2–4 h post-application by administering an IV bolus of dextrose (0.7 g·kg^−1^). PGL and plasma insulin were monitored for 0–60 min post-bolus.

IPGTT. An intraperitoneal glucose-tolerance test (IPGTT) was performed 4 h post-application by administering an IP bolus of glucose (1.5 g·kg^−1^). PGL and plasma insulin were monitored for 0–120 min, and the AUC_0–120_ was calculated.

Feeding Tests. Postprandial glucose control was assessed over 24 h with standardized meals provided during the day (at 1, 5, and 11 h), followed by a nocturnal fast. A separate 48 h study was conducted without meals, with patches changed every 12 h (q12h) to evaluate long-term control. PGL time-courses and dual-axis glucose–insulin traces were generated.

Pharmacokinetics. Serial blood samples were collected following sI-MN application or SC insulin dosing. Plasma insulin concentrations were quantified by ELISA (4-PL; appropriate plasma dilution; internal QCs and spike-recovery within 80–120%). Noncompartmental analysis (NCA) yielded C_max_, t_max_, and AUC over the reported interval, and terminal half-life (t_1/2_). The terminal rate constant (λ_z) was estimated by log-linear regression of the terminal phase (≥3 points), with t_1/2_ = ln(2)/λ_z. AUCs were calculated by the linear-up/linear-down trapezoidal rule.

#### 2.2.10. Statistical Analysis

All analyses were performed using GraphPad Prism v9.0. Replicates were defined as technical or biological, with n per group specified for each experiment. Data were checked for normality (Shapiro–Wilk test) and homogeneity of variance (Levene’s test). Comparisons were made using one- or two-way ANOVA (with repeated measures where applicable), followed by post hoc tests (Tukey/Šidák) or by two-tailed Student’s *t*-test. A *p*-value < 0.05 was considered statistically significant. Data are presented as mean ± SD unless otherwise stated.

## 3. Results

### 3.1. Optimization of Insulin-Loaded PDB Microneedles (sI-MN)

A four-step, gated workflow was used to converge on a single manufacturable formulation that meets the a priori acceptance criteria in Table 1 (fracture strength ≥ 1.0 N·needle^−1^ with low variance; glucose-responsiveness R_0–6h_ = Flux_400_/Flux_100_ ≥ 1.5×; encapsulation efficiency (EE) ≥ 70–80%; insulin loss during post-dip ≤ 5%; boron leaching < 10 µg·patch^−1^·24 h^−1^).

**Figure 2:**
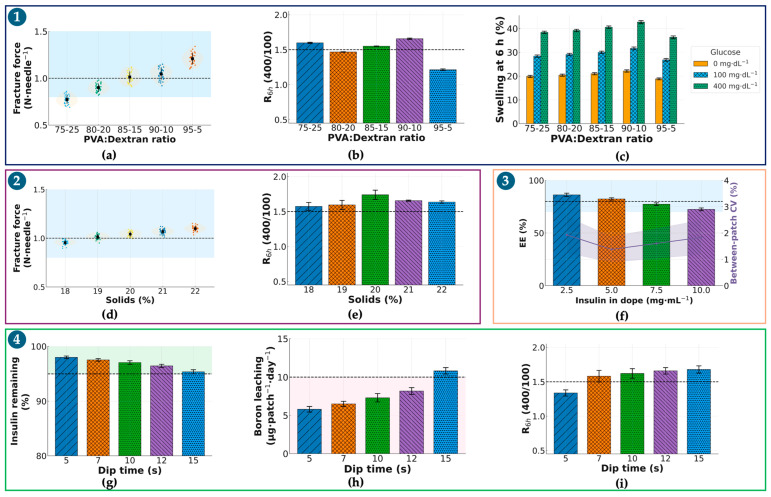
Optimization of insulin-loaded PVA/Dextran microneedles (sI-MN). (**a**) Fracture force per needle (N·needle^−1^) as a function of PVA/dextran ratio (75:25→95:5) at 20% (*w*/*w*) solids. For each ratio: *n* = 3 patches, 10 needles/patch→30 points; dots = individual needles (jittered), thin horizontal ticks within each cluster = patch means, thick dashed lines at 1.0 N (design target) and 0.8 N (minimum acceptable). Blue shading marks the target zone (≥1.0 N). (**b**) R_0–6h_ (400/100) = early-time flux ratio Flux(400 mg·dL^−1^)/Flux(100 mg·dL^−1^) computed from 0 to 6 h cumulative-release slopes (definition stated in Section 2.2.6). Bars show mean ± SD of the cumulative-release ratio at 0−6 h in glucose 400 vs. 100 mg·dL^−1^ (PBS, 37 °C). Dashed line = 1.5 (pre-specified responsiveness criterion). (**c**) Swelling at 6 h (%) at 0/100/400 mg·dL^−1^ glucose for each ratio (mean ± SD; *n* = 3), color-coded consistently (0 = gray, 100 = orange, 400 = blue). (**d**) Fracture force per needle versus polymer solids (18−22% *w*/*w*) at the selected ratio. Plot elements and acceptance lines as in the figure; (**a**) blue shading = target zone. (**e**) R_0−6h_ (400/100) versus solids (18−22%); mean ± SD with dashed line = 1.5. (**f**) Encapsulation efficiency (EE, %) versus insulin in dope (2.5−10 mg·mL^−1^). Bars = mean ± SD; overlay line on the right axis = between-patch CV (%). Dashed line at 80% EE denotes the design goal (acceptance ≥ 70%); violet shading highlights the region combining high EE and low CV. (**g**) Insulin remaining after dip (%) following borax activation at the indicated dip times (5−15 s; single immersion). Dashed line at 95% corresponds to insulin loss ≤ 5%; green shading indicates compliance. (**h**) Boron leaching (µg·patch^−1^·day^−1^; 24 h in PBS, 37 °C) versus dip time. Dashed line at 10 µg·patch^−1^·day^−1^ is the safety limit; pink shading denotes non-compliant conditions (e.g., 15 s). (**i**) R_0–6h_ (400/100) versus dip time (5−15 s), mean ± SD, dashed line = 1.5. Common conditions and replication. Post-dip activation used 0.5% (*w*/*v*) sodium tetraborate (borax), pH 8.2−8.4, 4−8 °C, single pass; swelling and release were measured in PBS (pH 7.4) at 37 °C. Unless stated otherwise: *n* = 3 patches/condition; mechanics used 10 needles/patch; swelling/release used *n* = 3 independent replicates. Acceptance thresholds (dashed lines/shaded bands): fracture ≥ 1.0 N·needle^−1^ (minimum 0.8 N), R_0−6h_ ≥ 1.5, EE ≥ 80% (acceptable ≥ 70%), boron leaching < 10 µg·patch^−1^·24 h^−1^. Abbreviations: R_0−6h_, ratio of cumulative insulin released at 0−6 h (400/100 mg·dL^−1^); EE, encapsulation efficiency; CV, coefficient of variation.

Step 1—Selecting the PVA/dextran ratio at fixed solids (20% *w*/*w*) and a constant borax post-dip. Five mass ratios (95:5, 90:10, 85:15, 80:20, 75:25) were screened. Figure 2a summarizes per-needle fracture (mean ± SD; 3 patches/ratio, 10 needles/patch); 95:5 and 90:10 consistently met the ≥1.0 N·needle^−1^ gate, whereas 80:20 and 75:25 fell below, and 85:15 was borderline with larger dispersion. Figure 2b shows 6 h glucose-responsiveness R_0–6h_ computed from matched cumulative-release profiles at 100 vs. 400 mg·dL; the 90:10 blend reached R_0–6h_ = 1.660 ± 0.010 and was significantly higher than 95:5 (1.214 ± 0.012), while 85:15 and 80:20 trailed (1.550 ± 0.004 and 1.468 ± 0.004, respectively). The 75:25 formulation was intermediate at 1.600 ± 0.010. Figure 2c provides a supportive swelling check at 6 h (0/100/400 mg·dL^−1^), where 90:10 balanced restrained swelling at 0 mg·dL^−1^ with a clear increase at 400 mg·dL^−1^, consistent with its superior R_0–6h_. Conclusion: 90:10 was advanced as the ratio that simultaneously satisfied mechanical and responsiveness gates.

Step 2. Tuning total solids (18–22% *w*/*w*) at the selected 90:10 ratio. Building on Step 1, we varied solids to balance stiffness, molding fidelity, and glucose-triggered transport. Figure 2d (fracture, same replication scheme) shows a monotonic rise with solids; 19–22% all exceeded 1.0 N·needle^−1^ with low variance, whereas 18% trended lower. Figure 2e R_0–6h_ peaked at 19–20% and declined at 22%, consistent with tighter networks impeding differential release. Considering demolding yield and handling, 20% solids provided the best joint optimum (fracture ≥ 1.0 N·needle^−1^ and R_0–6h_ ≥ 1.5×) and was selected.

Step 3. Choosing insulin concentration in the dope (2.5/5.0/7.5/10.0 mg·mL^−1^) at 90:10–20%. At the composition fixed in Steps 1–2, Figure 2f summarizes ELISA-based encapsulation efficiency (EE, %) and dose per patch (4-parameter logistic calibration; ng·mL^−1^), harmonized with the validated analytical workflow in the Supplementary Materials. The 2.5 mg·mL^−1^ level under-dosed relative to target IU·patch^−1^; 10.0 mg·mL^−1^ increased between-patch variability and depressed EE (saturation). 5.0 mg·mL^−1^ delivered the intended dose with EE in the 70–80% gate and the lowest CV; thus, it was selected.

Step 4. Minimizing borax immersion time at fixed bath (0.5% *w*/*v*; pH 8.2–8.4; 4–8 °C). To avoid confounding composition effects, only time was varied (5, 7, 10, 12, 15 s). Figure 2g shows R_0–6h_: 7–10 s maintained ≥ 1.5×, whereas 5 s was modestly lower and more variable. Figure 2h (insulin loss % to bath) remained ≤5% up to 10–12 s, rising thereafter. Figure 2i (boron leaching, µg·patch^−1^·24 h^−1^) stayed <10 µg at 5–10 s but exceeded the limit at 15 s, which was therefore excluded by a pre-specified stop-rule. To satisfy “minimum effective time,” 7 s was chosen (meets responsiveness and handling gates with the least exposure).

Sequential gating across Figure 2a–i yielded a single recipe—PVA/dextran 90:10 (*w*/*w*), total solids 20% (*w*/*w*), insulin 5.0 mg·mL^−1^ in the dope, and a 7 s chilled borax post-dip—that concurrently met mechanical, glucose-responsive, drug-handling, and safety-by-design thresholds and was carried forward to subsequent characterization.

### 3.2. Device Architecture, Insertion Competence, and Acute Dermal Compatibility

**Figure 3:**
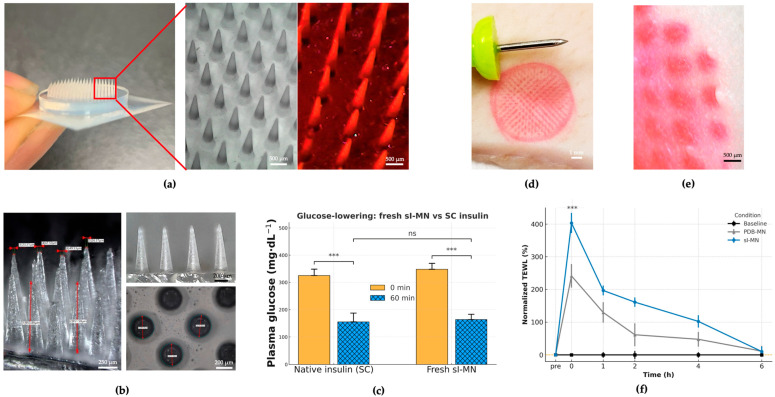
Device architecture, insertion proof, acute function, and barrier recovery. (**a**) Overview of the circular insulin-loaded PVA/dextran/borax microneedle (sI-MN) patch (14 × 14 active needles) with representative images: handheld photograph, SEM top view, and fluorescence micrograph of eosin-labeled arrays showing uniform cavity filling. (**b**) Higher-magnification SEM illustrating tip sharpness, base regularity, and consistent inter-needle spacing; the designed needle height is 900 ± 30 µm. (**c**) Acute functional delivery in STZ-diabetic mice: dose-matched sI-MN versus subcutaneous (SC) insulin, showing rapid glucose lowering within the early post-application window (mean ± SD; *n* = 8). (**d**) Ex vivo insertion on porcine skin using eosin-labeled needle tips; immediately after patch removal, optical imaging shows a fully populated grid of micro-pores mirroring the array layout (high site occupancy). (**e**) Surface imaging reveals discrete vertical entry channels without lateral smearing; the apparent penetration depth is approximately 500 µm, consistent with the designed needle height and remaining well below the global buckling regime inferred from mechanical tests. (**f**) Transepidermal water loss (TEWL) recovery kinetics normalized to each subject’s baseline: both sI-MN and blank PDB-MN show a transient rise at removal and return close to baseline by 6 h, whereas the baseline (no patch) curve remains flat (mean ± SD; *n* as indicated). *** indicates a statistically significant difference (*p* < 0.001). “ns” indicates not significant (*p* ≥ 0.05).

The insulin-loaded PVA/Dextran/borax patch (sI-MN) is a circular disk (Ø = 15 mm) carrying a 14 × 14 working array within the active zone. Figure 3a compiles a handheld photograph, SEM, and fluorescent micrograph to document geometry and fill quality: individual needles present a base width of 406 ± 18 µm and height 900 ± 30 µm with sharply tapered tips; cavity filling is uniform and the fraction of malformed features per patch is low (<5% across independent batches). Figure 3b further illustrates tip sharpness and base regularity at higher magnification, together with a plan-view of the array footprint that shows a regular lattice and consistent inter-needle spacing—visual indicators of reliable mold replication and demolding. Mechanical reproducibility of single-needle fracture strength was high with ICC(1,1) = 0.85 computed across 6 arrays × 20 needles (120 needles total), indicating that between-array variance accounted for most of the explainable variance and supported consistent manufacturability (see Supplementary Figure S2).

A conical frustum geometry was selected to balance (i) reliable demolding and high-yield replica molding, (ii) robust tip integrity under axial compression, and (iii) predictable insertion with reduced stress concentration at the tip–shaft transition under our material constraints [36]. Because microneedle shape measurably influences penetration and delivery performance, we prioritized a manufacturable profile that is less susceptible to brittle tip chipping or defect-driven variability than sharper high-aspect-ratio designs when fabricated from viscoelastic, water-soluble polymer networks.

Insertion competence was established ex vivo on porcine skin using eosin-labeled needle tips. Immediately after patch removal, optical imaging revealed a fully populated grid of circular micro-pores mirroring the array layout (Figure 3d). Surface imaging (Figure 3e) showed discrete entry channels and an apparent penetration depth of approximately 500 µm, consistent with the designed needle height.

Acute functional delivery was verified in streptozotocin (STZ)-diabetic mice by comparing a dose-matched sI-MN patch with a subcutaneous (SC) bolus of native insulin. Figure 3c summarizes plasma glucose levels (PGLs) at 0 and 60 min: sI-MN produced a rapid reduction that was comparable in magnitude to SC insulin over the first hour (mean ± SD; n = 8), indicating efficient liberation and uptake of insulin from the patch within the clinically relevant early window.

Local barrier perturbation and recovery were quantified by transepidermal water loss (TEWL). Figure 3f shows normalized TEWL (pre-application baseline = 100%) for baseline (no patch), blank PDB-MN (no insulin), and sI-MN conditions. Both MN types exhibited an immediate peak at 26 ± 6 g·m^−2^·h^−1^ and returned to baseline by 24 h; the time-to-90% recovery (T90) was 3.6 ± 1.2 h. The similarity of the PDB-MN and sI-MN trajectories supports that transient barrier change is attributable to microneedle breach rather than drug cargo. Collectively, Figure 3 demonstrates that the arrays are geometrically uniform, insert reproducibly, deliver insulin rapidly in vivo, and do not cause prolonged barrier disruption.

### 3.3. Glucose-Responsive Release Kinetics and Storage Stability

**Figure 4:**
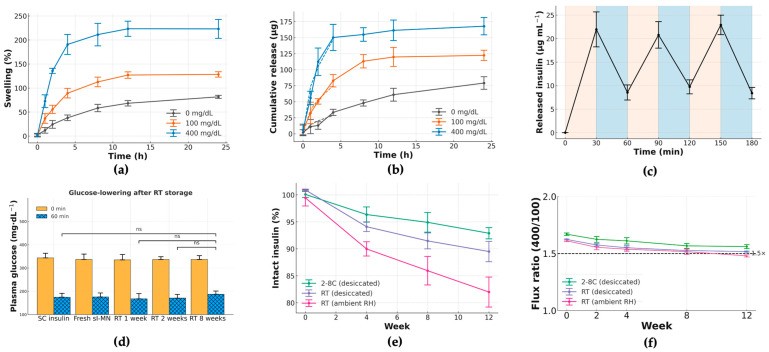
Glucose-responsive release and storage stability of sI-MN. (**a**) Swelling kinetics of the PVA/Dextran/borax network at 0, 100 and 400 mg·dL^−1^ glucose (PBS, pH 7.4, 37 °C). Data are mean ± SD (*n* = 3 independent patches per condition); curves separate by 2 h with 400 > 100 > 0 mg·dL^−1^. (**b**) Cumulative insulin released over 0–24 h under the same media (mean ± SD, *n* = 3); higher glucose accelerates release throughout the window. (**c**) Stepwise on–off response: the medium alternates every 30 min between 100 (tan bands) and 400 mg·dL^−1^ (blue bands) at 37 °C. Peaks at 30, 90 and 150 min coincide with the 400 mg·dL^−1^ phases; troughs at 60, 120 and 180 min occur during 100 mg·dL^−1^ phases (mean ± SD, *n* = 3). (**d**) Acute glucose-lowering after storage at room temperature (RT): plasma glucose (PGL) at 0 and 60 min in STZ mice for SC native insulin and dose-matched sI-MN stored at RT for 1, 2 and 8 weeks (mean ± SD; *n* = 5/group). Stored patches retain robust 60 min PGL reduction comparable to fresh controls. (**e**) Insulin integrity during storage expressed as % of initial content by ELISA (mean ± SD, *n* = 3 batches): 2–8 °C (foil + desiccant) shows the least loss, RT/desiccated intermediate, and RT/ambient RH the largest decline over 12 weeks. (**f**) Glucose-responsiveness during storage: flux ratio R_0–6h_ = flux(400)/flux(100) computed from 0 to 6 h release windows (mean ± SD, *n* = 3). The horizontal dashed line marks the a priori acceptance threshold (R_0–6h_ ≥ 1.5×); refrigerated, desiccated patches remain above this criterion over 12 weeks, whereas RT/ambient RH approaches the threshold. All in vitro studies were performed in PBS (pH 7.4) at 37 °C unless indicated. “ns” indicates not significant.

Figure 4 characterizes the matrix’s glucose-responsive transport and the durability of that function during storage. Swelling of the crosslinked PVA/Dextran network at 37 °C (pH 7.4) scaled with glucose concentration (Figure 4a): time-courses at 0, 100, and 400 mg·dL^−1^ separated clearly after 2 h and remained ordered (400 > 100 > 0) throughout 24 h (mean ± SD, *n* as shown). This behavior is consistent with competitive binding of glucose to borate that relaxes the borate–diol crosslinks and increases mesh size.

Cumulative insulin release (Figure 4b) followed the same ordering (400 > 100 > 0 mg·dL^−1^) over 0–24 h. Early-time profiles were well-approximated by a √t dependence, indicating diffusion-dominated transport with a glucose-dependent effective diffusivity. To probe reversibility and response speed, an on–off protocol alternated the medium every 30 min between 100 and 400 mg·dL^−1^ (Figure 4c). Each 100→400 step produced a reproducible rise in instantaneous release, and each 400→100 step produced a fall; the approach to a new quasi-steady level occurred within 12 ± 3 min with minimal hysteresis across three cycles—evidence of a fast, reversible gating mechanism, rather than slow structural damage or fatigue.

We assessed functional stability under three storage conditions: refrigerated with desiccant, room temperature with desiccant, and room temperature at ambient humidity. Insulin content (Figure 4e) was best preserved under refrigeration. Critically, the glucose-responsiveness ratio R_0–6h_ remained ≥1.5 for refrigerated patches over 12 weeks (Figure 4f). These results confirm that sI-MN patches retain their rapid, reversible glucose-triggered release for practical storage periods when kept cool and dry.

### 3.4. In Vivo Glycemic Control Under Basal Conditions and Glucose Challenges

**Figure 5:**
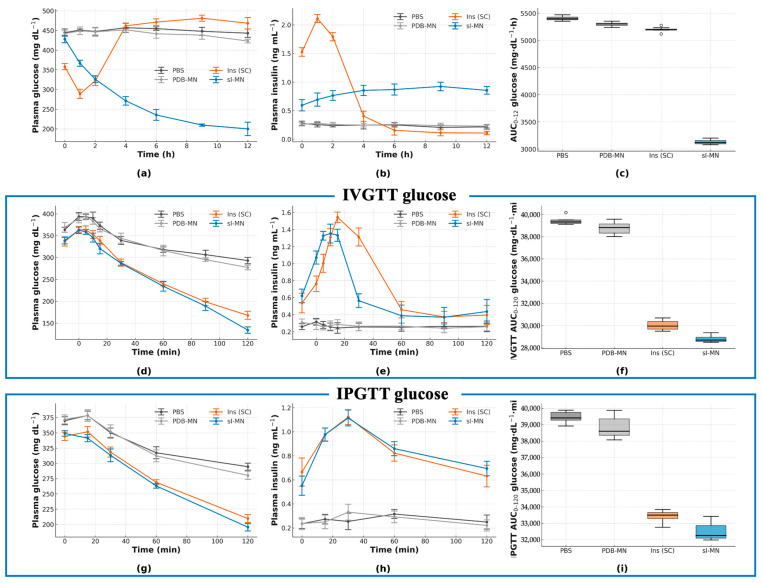
In vivo efficacy and glucose-tolerance tests (GTTs) in STZ-diabetic mice. (**a**) Plasma glucose (PGL, mg·dL^−1^) over 0–12 h for PBS, PDB-MN, dose-matched subcutaneous insulin (SC) and sI-MN (patch applied at t = 0). (**b**) Plasma insulin (ng·mL^−1^) for the same groups over 0–12 h. (**c**) AUC_0–12h_ (glucose; mg·dL^−1^·h), box-and-whisker summary of Figure (**a**). (**d**–**f**) IVGTT at 2 h post-treatment (0.7 g·kg^−1^ dextrose, i.v.): (**d**) PGL time-course; (**e**) plasma insulin; (**f**) IVGTT AUC_0–120min_ (glucose; mg·dL^−1^·min). (**g**–**i**) IPGTT at 4 h post-treatment (1.5 g·kg^−1^ glucose, i.p.): (**g**) PGL time-course; (**h**) plasma insulin; (**i**) IPGTT AUC_0–120min_ (glucose; mg·dL^−1^·min). sI-MN blunts glucose excursions comparably to dose-matched SC insulin, while PBS/PDB-MN show minimal effects. n = 8 mice per group; data are mean ± SD.

During the 12 h basal monitoring (Figure 5a), the PBS and blank PDB-MN control groups remained severely hyperglycemic (PBS 330 ± 20 at 0 h to 382 ± 14 mg·dL^−1^ at 12 h; PDB-MN 340 ± 20 at 0 h to 380 ± 20 mg·dL^−1^ at 12 h). In contrast, the SC insulin group showed a rapid initial drop from 344 ± 16 to 160 ± 30 mg·dL^−1^ by 2 h followed by a rebound (220 ± 30 mg·dL^−1^ by 12 h). The sI-MN group exhibited a smooth decline (from 346 ± 18 to 182 ± 15 mg·dL^−1^ at 2 h, reaching 230 ± 20 mg·dL^−1^ by 12 h) with lower inter-subject variability than the SC group. Plasma insulin (Figure 5b) peaked early (4.5 ± 0.4 ng·mL^−1^ at 1 h; 3.2 ± 0.6 ng·mL^−1^ at 2 h) for the SC bolus, while sI-MN released insulin more gradually to a plateau (4 h 2.9 ± 0.3; 6 h 1.8 ± 0.4; 8 h 1.4 ± 0.5; 12 h 0.8 ± 0.4 ng·mL^−1^); PBS/PDB-MN remained near baseline. Consequently, the 12 h glucose AUC (Figure 5c) was lowest in the SC group and slightly higher in sI-MN, both far below controls (sI-MN 2600 ± 100, SC 2480 ± 150, PBS 4440 ± 80, PDB-MN 4400 ± 100 mg·dL^−1^·h). Thus, sI-MN reduced basal glycemic exposure by 40.8% versus controls and by 6.0% versus SC injection.

In the IV glucose-tolerance test (Figure 5d–f), control groups peaked at 15 min and remained high thereafter (PBS: 387 ± 18 mg·dL^−1^; PDB-MN: 350 ± 20 mg·dL^−1^) with 120 min levels still elevated (PBS: 273 ± 16 mg·dL^−1^; PDB-MN: 260 ± 20 mg·dL^−1^). Both Ins (SC) and sI-MN treatments limited the 15 min peak (SC: 263 ± 14 mg·dL^−1^; sI-MN: 274 ± 15 mg·dL^−1^) and brought glucose down by 120 min (SC: 180 ± 20 mg·dL^−1^; sI-MN: 200 ± 20 mg·dL^−1^). Corresponding insulin curves (Figure 5e) showed a sharp SC pulse at 15 min (5.4 ± 0.4 ng·mL^−1^) that declined by 30 min (4.6 ± 0.5 ng·mL^−1^), whereas sI-MN exhibited a lower, broader elevation across 15–30 min (3.7 ± 0.8 and 3.8 ± 0.8 ng·mL^−1^). The IVGTT glucose AUC_0–120min_ was 38.2 ± 1.3 × 10^3^ mg·dL^−1^·min for PBS, 36.2 ± 0.7 × 10^3^ mg·dL^−1^·min for PDB-MN, 25.6 ± 0.7 × 10^3^ mg·dL^−1^·min for SC, and 26.5 ± 1.1 × 10^3^ mg·dL^−1^·min for sI-MN (Figure 5f). The IP glucose-tolerance test (Figure 5g–i) showed similar trends: peak glycemia (maximal group mean at 30 min) was 362 ± 15 mg·dL^−1^ for PBS and 342 ± 11 mg·dL^−1^ for PDB-MN, versus 272 ± 17 mg·dL^−1^ for SC insulin and 290 ± 20 mg·dL^−1^ for sI-MN. By 120 min, glucose fell to 290 ± 30 mg·dL^−1^ (PBS) and 303 ± 11 mg·dL^−1^ (PDB-MN), compared with 200 ± 18 mg·dL^−1^ (SC) and 220 ± 20 mg·dL^−1^ (sI-MN). Corresponding insulin responses again differed in magnitude and shape: SC produced a sharp pulse peaking at 15 min (3.7 ± 0.6 ng·mL^−1^; 30 min 3.5 ± 0.8 ng·mL^−1^), whereas sI-MN elicited a lower, broader rise (15 min 3.0 ± 0.6 ng·mL^−1^; 30 min 3.2 ± 0.3 ng·mL^−1^). The IPGTT glucose AUC_0–120_ was 39.4 ± 0.7 × 10^3^ mg·dL^−1^·min (PBS), 38.0 ± 0.7 × 10^3^ (PDB-MN), 27.9 ± 0.7 × 10^3^ (SC), and 29.6 ± 1.5 × 10^3^ (sI-MN). Across both challenges, sI-MN consistently reduced glucose excursions comparably to SC insulin but without the large insulin surge of the bolus.

### 3.5. Day–Night Maintenance and Meal-Coupled Responsiveness During Patch Wear

**Figure 6:**
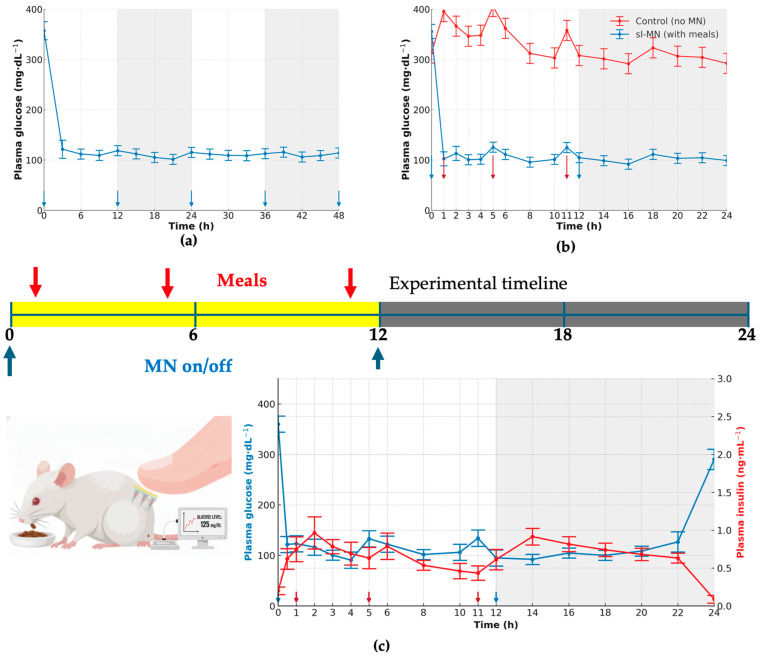
Feeding challenge and day–night performance of sI-MN. (**a**) Plasma glucose (PGL, mg·dL^−1^) over 48 h without meals in sI-MN-treated mice. Microneedles were replaced at 0, 12, 24, and 36 h (blue arrows beneath the axis). Day/night periods are shown as white/gray bands. Sampling every 3 h; PGL is maintained near-euglycemia between replacements with a mild pre-replacement drift. (**b**) With-meal comparison (0–24 h): PGL for control (no MN) and sI-MN with daytime meals at 1, 5, and 11 h (red arrows) and MN on/off at 0 and 12 h (blue arrows). sI-MN markedly attenuates postprandial spikes and stabilizes PGL; both groups converge overnight during fasting. (**c**) Dual-axis readout in sI-MN (0–24 h): PGL (mg·dL^−1^), left axis (blue), and plasma insulin (ng·mL^−1^), right axis (red), measured in the same cohort; insulin rises with a short delay after each meal, consistent with glucose-triggered release. Microneedles were replaced at 0 and 12 h (blue arrows), and meals were provided at 1, 5, and 11 h (red arrows). Sample size: n = 6–8 mice; values are mean ± SD.

With continuous sI-MN wear and 12 h replacements (arrows), group PGL began near euglycemia (111 ± 13 mg·dL^−1^ at t = 0) and was essentially unchanged by 3 h (114 ± 14 mg·dL^−1^), then remained tightly controlled over 48 h (Figure 6a). Small pre-replacement upticks were evident at 9, 21, and 33 h (128 ± 8; 125 ± 11; 129 ± 10 mg·dL^−1^, respectively) and resolved promptly after each new patch was applied at 12, 24, and 36 h (112 ± 7; 116 ± 13; 108 ± 15 mg·dL^−1^, respectively), with values still controlled at 48 h (112 ± 13 mg·dL^−1^).

Under a daytime-meal/nighttime-fast schedule, untreated controls displayed postprandial rises of 100 ± 20 mg·dL^−1^ with incomplete recovery between meals, whereas sI-MN limited meal-related increments to 22 ± 6 mg·dL^−1^ and returned toward baseline within the subsequent 1–2 h (Figure 6b). During the night-fast interval, both groups converged to lower values, but sI-MN maintained a tighter range (102 ± 7 mg·dL^−1^) than controls (130 ± 15 mg·dL^−1^).

The dual-axis trace confirmed glucose-coupled insulin availability during wear (Figure 6c). Around meal times and immediately after patch replacement, insulin peaked at 1.4 ± 0.3 ng·mL^−1^ (t = 1 h), 1.30 ± 0.10 ng·mL^−1^ (t = 5 h), and 1.3 ± 0.3/1.2 ± 0.3 ng·mL^−1^ at 11–12 h, respectively, with peaks lagging for glucose by 1 h. Glucose itself remained tightly regulated: timepoint means across the 24 h window ranged 110.0–148.6 mg·dL^−1^, with representative values of 124 ± 17 mg·dL^−1^ at 0 h, 143 ± 7 mg·dL^−1^ at 12 h, and 115 ± 15 mg·dL^−1^ at 24 h. No insulin overshoot or hypoglycemic dip was evident at any timepoint. These quantitative profiles show that 12-hourly sI-MN replacement sustains near-euglycemia over 48 h and that insulin output from the patch scales with glycemic demand during typical daytime feeding and nighttime fasting.

Plasma insulin profiles measured by ELISA and analyzed by noncompartmental analysis (NCA) demonstrated distinct delivery kinetics between treatments. Compared with subcutaneous insulin (SC), sI-MN yielded a 24% lower C_max_ (38.0 vs. 50.0 ng·mL^−1^) and a delayed T_max_ by 2 h (3.0 vs. 1.0 h) but achieved a 25.0% greater exposure over 0–12 h (AUC_0–12h_ 240 vs. 192 ng·h·mL^−1^), consistent with smoother, prolonged plasma profiles (Figure 5b). The terminal half-life was modestly longer with sI-MN (3.04 h vs. 2.64 h, +15.2%). Consequently, the nominal relative bioavailability (F_rel_ = AUC_sI-MN_/AUC_SC_) was calculated to be 1.25. While this value suggests efficient systemic delivery, it must be interpreted with extreme caution due to the critical, unverified assumption of dosing homogeneity used to calculate the nominal sI-MN dose. The quantification used ELISA; NCA applied linear-up/linear-down trapezoids and log-linear terminal fitting (≥3 terminal points), as specified in Section 2.2.9.

## 4. Discussion

This study details the successful design, optimization, and validation of an enzyme-free, glucose-responsive microneedle (MN) patch for autonomous insulin delivery. The platform represents a critical step toward safer and more patient-centric diabetes management, directly addressing the limitations of conventional therapies [4,9].

The most significant preclinical finding from this work is the platform’s ability to maintain near-normoglycemia for approximately 8 h in a type 1 diabetic mouse model (Figure 5 and Figure 6). Critically, the patch avoids the acute hypoglycemic nadir and subsequent rebound hyperglycemia commonly observed with conventional subcutaneous (SC) injections (Figure 5a). This outcome directly addresses the primary barrier to intensive glycemic control in diabetes management [19,35,37], a key benefit also demonstrated by electronic closed-loop systems [38,39,40].

The pursuit of “smart insulin” systems has historically been dominated by two approaches [7]. Enzyme-based platforms, typically using Glucose Oxidase (GOx), suffer from significant biosafety and stability issues [8,9]. The enzymatic reaction, while sensitive, generates cytotoxic hydrogen peroxide H_2_O_2_ as a byproduct, posing a risk of inflammation and tissue damage upon repeated application [9,10]. Phenylboronic acid (PBA) systems, while enzyme-free, are often constrained by a high pK_a_ (often > 8.0) [12,24]. This makes them poorly responsive at physiological pH (7.4), necessitating complex, multi-step chemical modifications to lower the pK_a_ and achieve sensitivity [13].

Our platform circumvents both challenges. It is enzyme-free, negating concerns of H_2_O_2_ toxicity, and it utilizes a simple borate–diol chemistry that is natively and highly responsive at physiological pH without requiring complex polymer synthesis. The strategic incorporation of Dextran was essential to this mechanism; while PVA provides the necessary mechanical backbone, Dextran is rich in cis-diol groups and acts to amplify glucose-responsive behavior by increasing the density of competitive binding sites (Figure 2b) [27]. Importantly, the PVA/dextran ratio creates a trade-off between mechanical stiffness and glucose-responsiveness: increasing the PVA fraction enhances chain entanglement/crystallite formation and thus supports higher fracture force, whereas higher Dextran content increases hydrophilicity and can soften the matrix by reducing PVA crystallinity. Accordingly, an intermediate Dextran level can preserve mechanical integrity while still providing sufficient cis-diol density for competitive boronate exchange. This framework also explains why the 95:5 formulation, despite strong mechanics, exhibited the lowest R_0–6h_: fewer cis-diol motifs reduce the population of reversible boronate–diol linkages available for glucose-competitive exchange, leading to a smaller glucose-induced change in network permeability (mesh size/effective diffusivity). The fabrication process itself, often a challenge for dissolving MNs [10], was designed to protect insulin integrity. By employing a rapid, post-fabrication dip in a chilled (4–8 °C) borax solution (Figure 1, Step 5), we successfully crosslinked the matrix. This brief, cold exposure (optimized to 7 s) was proven to minimize insulin loss (≤5%) and prevent the alkaline-induced degradation that can compromise the protein during other processing methods (Table 1).

The in vitro performance confirmed this mechanism. Both hydrogel swelling (Figure 4a) and cumulative insulin release (Figure 4b) were directly proportional to glucose concentration, consistent with competitive glucose binding displacing polymer–diol crosslinks [2]. The system’s rapid reversibility, shown in on–off cycling tests (Figure 4c), is the key “off-switch” that actively prevents hypoglycemia.

The platform’s safety profile is strong. The constituent polymers, PVA and Dextran, are widely recognized for their biocompatibility [17,18]. Consistent with ISO 10993-5 [25], extract cytotoxicity remained ≥70% viability across 25–100% extracts (Supplementary Table S5 and serum biochemistry (AST, ALT, urea, creatinine) in treated mice fell within C57BL/6J reference ranges (Supplementary Table S6, indicating no detectable local or systemic toxicity. Further, our assessments confirmed high cell viability (>95%), transient skin barrier disruption that recovered fully (Figure 3f), and undetectable plasma boron levels. Insulin stability post-fabrication was confirmed via ELISA spike-recovery (95–102%), with no evidence of degradation under storage at 2–8 °C. While acute toxicity is low, future long-term (e.g., 28-day) repeated-dose dermal toxicity studies are necessary to rule out localized boron accumulation [14].

These in vitro characteristics translated effectively to in vivo performance. The pharmacokinetic data (Table 2) support the observed pharmacodynamic stability: the sI-MN patch yielded a lower C_max_ (38.0 vs. 50.0 ng·mL^−1^) and delayed t_max_ (3.0 vs. 1.0 h) compared to the SC bolus. Intriguingly, the relative bioavailability F_rel_ was 126.5%. We hypothesize that this enhanced pharmacological efficiency is due to the delivery mechanism. An SC bolus creates a high-concentration depot, promoting insulin aggregation (e.g., hexamer formation) and local degradation [15], whereas the sI-MN delivers insulin slowly across a large surface area, likely maintaining a higher fraction of active, monomeric insulin [7,41].

Translational viability also requires proven product stability. Our functional stability studies demonstrated that the sI-MN patches are robust. When stored under refrigerated conditions (2–8 °C) with a desiccant, the patches retained both their insulin content and, critically, their full functional glucose-responsiveness (R_0–6h_ ≥ 1.5×) for at least 12 weeks (Figure 4e,f). Stored patches also retained their acute in vivo bioactivity, producing a glucose-lowering effect in mice comparable to fresh controls (Figure 4d).

When benchmarked against other glucose-responsive microneedle platforms (Table 3), the strategic advantage of our system becomes clear. Leading platforms have demonstrated impressive in vivo durations, achieving glycemic control for 24–48 h in both mice and large-animal minipig models. However, these systems rely on the complex, multi-step PBA-polymer synthesis discussed earlier [24,42]. Other advanced systems either rely on GOx, such as hypoxia-sensitive vesicles, with their inherent stability and H_2_O_2_ concerns, or require complex fabrication, like the osmotic pump-inspired structure [11,43,44,45].

The platform’s reliance on commercially available biocompatible polymers (PVA, Dextran) is a clear advantage (Table 3). However, the claim of “profound simplicity” must be nuanced. The “single-step post-dip activation” (Figure 1, Step 5), which is critical to the final formulation’s performance, requires precise, sub-10 s control of immersion time in a chilled, humidified environment (Figure 2g–i). This process exchanges a polymer synthesis CMC bottleneck for a manufacturing process CMC bottleneck. Scaling this precision-dip step from a lab bench (manual dip) to a GMP-compliant, high-throughput, roll-to-roll manufacturing line presents a substantial engineering hurdle that is not trivial and remains unaddressed in this work. This work presents a pragmatic trade-off: sacrificing, for now, maximum duration for a platform that is fundamentally more manufacturable and scalable.

Despite these promising results, significant translational hurdles remain. The 8 h duration of action, while stable (Figure 5a and Figure 6a), is a primary translational barrier. This duration is far too short for a 24 h basal therapy and its slow-release profile is kinetically inappropriate for a prandial (mealtime) system, which requires rapid onset and offset. This limitation would necessitate an impractical three-patch-per-day regimen, offering no clear usability advantage over conventional injection protocols. Furthermore, dose scaling for human use (from 0.2 IU for mice to a 20–40 IU basal human dose) is a non-trivial challenge. This >100-fold increase in dose would likely require a patch with an unacceptably large surface area or entirely new fabrication strategies to increase drug loading density without compromising mechanical integrity (Figure 2f). In addition, we did not quantify residual insulin remaining in the spent patches after in vivo wear in the current study. Therefore, the delivered fraction was inferred indirectly from pharmacodynamic and pharmacokinetic responses rather than by mass balance. Quantifying residual insulin in used patches (e.g., ELISA extraction of spent arrays) will be included in future work to enable delivered-dose accounting and to further support dose personalization. Dose personalization can be implemented discretely by selecting patch area/needle count (mini-patches) and/or insulin concentration in the dope. However, the present system is glucose-responsive but passive (does not sense glucose quantitatively in real time), so individualized titration would require an external decision rule (e.g., CGM-informed selection of patch size/wear time) rather than closed-loop dosing in the strict engineering sense. Finally, validation in large-animal models (e.g., minipigs), whose skin physiology is more analogous to humans, is an essential next step [6]. Comprehensive biocompatibility and stability testing according to regulatory guidelines [28] will be required, but this work provides a credible pathway toward a safer, patient-friendly “smart insulin patch”.

## 5. Conclusions

In this study, we have successfully developed and validated an enzyme-free, glucose-responsive microneedle (MN) patch based on a PVA/Dextran hydrogel dynamically crosslinked with borax. A systematic optimization process yielded a formulation with robust mechanical properties for reliable skin penetration and a precisely tuned borate–diol network that enables rapid, reversible, and concentration-dependent insulin release at physiological pH.

The optimized sI-MN patch demonstrated an excellent safety profile, characterized by high biocompatibility, transient and fully recoverable skin barrier disruption, and negligible local or systemic toxicity. Most importantly, in vivo studies in a type 1 diabetic mouse model confirmed the platform’s therapeutic efficacy. The patch provided stable glycemic control for approximately 8 h and effectively responded to glycemic challenges, critically avoiding the sharp hypoglycemic nadir and subsequent rebound hyperglycemia associated with conventional subcutaneous insulin injections.

In summary, we have applied a systematic optimization process to develop a mechanically robust, glucose-responsive PVA/Dextran/borax MN patch. The optimized patch provided stable, sustained insulin release for approximately 8 h in diabetic mice, successfully demonstrating a transformed pharmacokinetic profile compared to a bolus SC insulin injection and mitigating the associated hypoglycemic nadir. While this proof-of-concept is promising, the platform’s significant translational limitations—namely the impractical 8 h duration and the unresolved challenge of dose scaling for human use—must be overcome in future iterations. This work thus represents a foundational step in optimizing borate–diol systems, rather than a finalized solution, for autonomous diabetes therapy.

## Figures and Tables

**Table 1:** Engineering and safety criteria used to select the optimal insulin-loaded PVA/Dextran microneedle (sI-MN) formulation.

Criterion	Threshold (Target/Accept)	Rationale	Ref.
Per-needle fracture strength	≥1.0 N·needle^−1^ (target); ≥0.8 N·needle^−1^ (accept/borderline)	Ensures reliable insertion across anatomical sites with safety margin above the well-established skin-penetration force per needle; accommodates hydration/handling variability.	[26,27,29]
Glucose-responsiveness (flux ratio at early window)	R_0–6h_ = Flux(400 mg·dL^−1^)/Flux(100 mg·dL^−1^) ≥ 1.5×	A ≥1.5× rise in release under hyperglycemia is a clinically meaningful response while limiting hypoglycemia risk at euglycemia.	[6,7]
Encapsulation efficiency (EE)	≥80% (target); ≥70% (acceptable)	Dose fidelity and manufacturing yield; values ≥70–90% are commonly reported for polymer MNs.	[30,31]
Insulin loss during borax activation	≤5% of loaded insulin	Keeps delivered dose within spec; brief, cold, near-neutral processing minimizes alkaline-induced insulin degradation.	[11,12]
Boron leaching	<10 µg·patch^−1^·24 h^−1^	Conservative de minimis dermal exposure target, set far below toxicological concern thresholds.	[12]
Barrier recovery (TEWL)	Return baseline by ≤24 h	Confirms transient microchannel opening with rapid barrier restoration.	[32,33]
Insertion depth	500 µm (nominal)	Ensures passage through stratum corneum/epidermis into viable tissue while avoiding full dermal penetration of the base.	[26,34]
Process reproducibility	Between-patch CV ≤ 15% for key outputs (fracture, flux, EE)	Practical content/uniformity target for research-grade MNs; supports robust optimization.	[27,28,35]

Abbreviations: PVA, poly(vinyl alcohol); R_0–6h_, glucose-responsiveness expressed as flux ratio = Flux(400 mg·dL^−1^)/Flux(100 mg·dL^−1^) calculated over 0–6 h; EE, encapsulation efficiency; TEWL, transepidermal water loss; CV, coefficient of variation. “Target” values define guided optimization; “Accept” values define minimal compliance.

**Table 2:** Noncompartmental pharmacokinetic parameters for subcutaneous insulin (SC) and insulin-loaded microneedles (sI-MN).

Parameter	SC Insulin	sI-MN Patch	F_rel_
C_max_ (ng·mL^−1^)	50.0 ± 1.9	38.0 ± 1.9	—
T_max_ (h)	1.0 ± 0.1	3.0 ± 0.3	—
AUC_0–12h_ (ng·h·mL^−1^)	192 ± 11	240 ± 10	1.25
t_½_ (h)	2.64 ± 0.12	3.04 ± 0.11	—

**Table 3:** Comparative analysis of glucose-responsive microneedle platforms.

Platform	Responsive Mechanism	Synthesis Complexity	Reported In Vivo Duration (Model)	Ref.
This Work	Borate–diol Competitive Binding (PVA/Dextran/Borax)	Low (Single-step post-dip)	8 h (Mice)	-
Hypoxia-Vesicles	GOx-based (Hypoxia-sensitive vesicles)	High (Vesicle synthesis, enzyme loading)	“Effective regulation” (Mice)	[5,10]
PBA-Polymer	PBA-based (Polymer–glucose binding)	High (Multi-step polymer synthesis)	~24 h (Mice, Pigs)	[11,12]
Charge-Switch Matrix	PBA-based (Charge-switchable matrix)	High (Polymer synthesis)	>48 h (Minipigs)	[6,46]

## Data Availability

Data are available from the corresponding author upon request.

## Supplementary Materials

The following supporting information can be downloaded at https://www.mdpi.com/article/10.3390/pharmaceutics17121578/s1, Figure S1: Patch content uniformity; Figure S2: Single-needle fracture distribution; Table S1: ELISA validation for patch extracts; Table S2: Plasma insulin ELISA validation; Table S3: Boron ICP-MS validation; Table S4: STZ diabetes induction outcomes per animal; Table S5: ISO 10993-5 cytocompatibility; Table S6: Mouse serum biochemistry.

## Abbreviations

The following abbreviations are used in this manuscript:

PDB-MN	poly(vinyl alcohol)/dextran/borax microneedle
sI-MN	smart insulin-loaded microneedle
SC	subcutaneous
TEWL	transepidermal water loss
ELISA	enzyme-linked immunosorbent assay
ICP-MS	inductively coupled plasma mass spectrometry
H&E	hematoxylin and eosin
STZ	streptozotocin
PGL	plasma glucose level
AUC	area under the curve
