# Supplementary material for: Systematic Development and Characterization of Enzyme-Free, Borax-Crosslinked Microneedles for Glucose-Responsive Insulin Delivery and In Vivo Glycemic Mitigation
Source: Pharmaceutics. 2025 Dec 8;17(12):1578. doi: 10.3390/pharmaceutics17121578 (PMC12737151; doi:10.3390/pharmaceutics17121578)
Supplement: Supplementary file 1 [file pharmaceutics-17-01578-s001.zip › pharmaceutics-4002847-supplementary.pdf]

**Supplementary Information**

*Article*

# Systematic Development and Characterization of Enzyme-Free, Borax-Crosslinked Microneedles for Glucose-Responsive Insulin Delivery and *In Vivo* Glycemic Mitigation

**Cuc Thi Dinh[1], Linh Phuong Nguyen[2], Uyen Thu Pham[3], Anh Mai Nguyen[3], Hanh Thi My Do[3], Toan Quoc Tran[1 5], Phuong Duc Luu[1], Tien Duy Doan[1], Mo Thi Hong Bui[1], and Duong Thanh Nguyen[4 5]***

## Materials

Poly(vinyl alcohol) (PVA, 98–99% hydrolyzed, Mw 85–124 kDa), Dextran (40–70 kDa) and Eosin Y were purchased from Sigma-Aldrich (St. Louis, MO, USA). Phosphate-buffered saline (PBS, pH 7.4) were obtained from Thermo Fisher Scientific (Waltham, MA, USA). Sodium tetraborate decahydrate (borax) were sourced from Merck KGaA, (Darmstadt, Germany). For patch extracts and in-vitro release samples, human insulin ELISA (Mercodia AB, Uppsala, Sweden; Cat. 10-1113-01) was used and quantified in $ng \cdot mL^{-1}$ with a 4PL calibration (typical 0.30–10 $ng \cdot mL^{-1}$). For plasma PK, human insulin ELISA (Mercodia AB, Uppsala, Sweden; Cat. 10-1132-01) was used with the same unit system ($ng \cdot mL^{-1}$) to harmonize reporting; manufacturer cross-reactivity to mouse insulin <0.1% and our spike-recovery 95–102% confirmed selectivity. For ICP-MS, trace-metal-grade nitric acid ($HNO_3$) was from Fisher Chemical, Thermo Fisher Scientific (Waltham, MA, USA). Ultrapure water (18.2 $M\Omega \cdot cm$) was prepared on site. Unless specified, experiments were conducted at ambient laboratory temperature (20–25 °C) with at least three independent replicates, and analytical acceptance followed common bioanalytical criteria (calibration $R^2 \geq 0.99$; QC bias 80–120%; QC CV ≤ 15%—≤20% at LLOQ; carryover <20% of LLOQ; dilution-linearity/parallelism within ±15%).

## Methods

### *Patch Content Uniformity*

Whole patches were fully dissolved in PBS (pH 7.4, 37 °C) and filtered (0.22 µm). Insulin content was quantified by human insulin ELISA with a 4-parameter logistic (4PL) calibration curve (≥5 non-zero standards; $R^2 \geq 0.99$). Quality controls met 80–120% accuracy with intra-/inter-assay precision CV ≤

15%. Results are reported as insulin IU per patch and CV% across production patches (related to Section 2.2.3).

*Mechanical Robustness — Single-Needle Fracture*

Six independent arrays (n = 20 needles per array) were tested under micro-compression at constant speed. Fracture point was identified from force–displacement traces. Per-array mean ± SD and overall CV were computed. Reproducibility across arrays was summarized by a one-way random-effects ICC(1,1) (related to Section 2.2.4).

*ELISA Validation — Patch Extract Matrices*

This assay quantifies insulin in fully dissolved microneedle (MN) patches (content and content uniformity) and in in-vitro release samples. For content, each full-size patch was dissolved in PBS (pH 7.4) at 37 °C with gentle shaking for 30–60 min, clarified through a 0.22 μm membrane, and diluted ≥1:10 in ELISA sample diluent to fall within the validated range. For in-vitro release, time-point aliquots were immediately diluted ≥1:10 in ELISA diluent and assayed the same day; if storage was necessary, samples were kept on ice during handling and frozen at −80 °C for no more than one freeze–thaw before analysis. Calibration employed five to seven non-zero standards (typical range 0.30–10 $ng \cdot mL^{-1}$) fitted by a four-parameter logistic (4PL) model with $1/y^2$ weighting. The LLOQ was defined as the lowest level meeting bias and CV ≤20%, and the ULOQ as the highest level meeting bias and CV ≤15%. Low/Mid/High QCs and a matrix-matched QC (blank patch extract spiked with insulin) were included in duplicate; unknowns were run in duplicate with corner wells avoided to minimize edge effects. Carryover was checked by placing an LLOQ well immediately after the ULOQ well. Matrix effect and dilution parallelism were verified by serial dilutions (e.g., 1:5–1:80) requiring back-calculated concentrations within ±15% and slopes within 0.85–1.15 relative to standards. A run was accepted if ≥2/3 QC levels were within 80–120% recovery and CV ≤15% (≤20% at LLOQ). Content (IU per patch) was calculated from back-calculated insulin mass using 1 IU = 0.0347 mg (34.7 μg) human insulin (≈28.8 $IU \cdot mg^{-1}$). All ELISA-derived masses were converted accordingly before IU normalizatio. Uniformity was summarized as mean ± SD, CV%, and min–max (e.g., n = 24 patches). Release was expressed as cumulative percent released over time; flux (0–6 h) was obtained from early-time linear fits, and on–off cycling metrics included $R_{0\text{–}6}$h = Flux(400)/Flux(100) and T90 (time to reach 90% of the new plateau).

*ELISA Validation — Mouse Plasma (PK)*

This method quantified human insulin in mouse plasma for pharmacokinetic (PK) analyses. Serial blood samples were collected into $K_2$-EDTA tubes, gently inverted, and kept on wet ice. Plasma was separated at 2–4 °C (1,500–2,000 g, 10 min), aliquoted, and stored at −80 °C until analysis; hemolyzed samples (pre-specified visual criterion) were excluded. Samples were diluted 1:5–1:20 in kit diluent. Five to seven non-zero standards (typical 0.30–10 $ng \cdot mL^{-1}$) and QCs were run in duplicate and fitted by 4PL ($1/y^2$). Selectivity against endogenous mouse insulin was ensured per manufacturer documentation (cross-reactivity

<0.1%) and confirmed by blank plasma < LLOQ. Stability was verified for bench-top (6 h ≥95%), three freeze–thaw cycles (≥90%), and −80 °C storage for 14 days (≥90%). PK outputs per animal included observed Cmax, Tmax, $AUC_{0-12h}$ (linear-up/linear-down trapezoids), $\lambda z$ from ≥3 terminal points (log-linear regression), and t½ = ln2/$\lambda$z. Group means ± SD are reported in Table 2 of the main text; individual per-mouse tables are provided to the journal as source data at submission.

*ICP–MS Validation — Boron Leaching*

MN patches (n ≥ 3) were incubated in PBS (pH 7.4) at 37 °C for 24 h at a defined liquid-to-patch ratio (e.g., 2 mL per patch) in acid-washed polypropylene tubes. Leachates were immediately acidified to 1% $HNO_3$ (trace-metal grade, Fisher Chemical, Thermo Fisher Scientific (Waltham, MA, USA), stored at 4 °C, and analyzed within 24–48 h. ICP-MS operated in no-gas mode monitoring $^{11}B$ (and optionally $^{1}$[illegible]B), with an online internal standard (e.g., $^{115}In$ 10 μg·L$^{-1}$, Inorganic Ventures (Christiansburg, VA, USA). External calibration used 0–100 μg·L$^{-1}$ standards (matrix-matched), with $R^2 \geq 0.99$ required. A rinse of 2% $HNO_3$ for ~30 s was applied between injections to mitigate boron memory. Continuing calibration blank (CCB) and verification (CCV) were run approximately every ten injections (bias ≤ ±10%), and instrumental drift was required to remain ≤5% over the batch. LOD and LOQ were estimated from low-level blanks and calibration residuals (typical LOD ~0.20 μg·L$^{-1}$; LOQ ~0.60 μg·L$^{-1}$). Low/Mid/High QCs required 80–120% recovery with CV ≤ 15%. Concentrations (μg·L$^{-1}$) were converted to μg·patch$^{-1}$·day$^{-1}$ using the leachate volume and number of patches; the acceptance criterion for boron leaching was <10 μg·patch$^{-1}$·24 h$^{-1}$.

*STZ Diabetes Induction*

Streptozotocin (STZ) was prepared fresh daily in 0.1 M citrate buffer (pH 4.5) on ice, protected from light, and injected intraperitoneally at 55 mg·kg$^{-1}$ once daily for five consecutive days. Animals were fasted for 6–8 h prior to each STZ dose and prior to glycemic assessments as applicable. Fasting plasma glucose (FPG) was measured seven days after the last STZ dose using a calibrated glucometer. Mice with FPG > 300 mg·dL$^{-1}$ were considered diabetic and enrolled; animals not meeting the FPG criterion or reaching humane endpoints were excluded per pre-specified rules. All animal procedures complied with institutional oversight (protocol ILES-IACUC-2025-002; approved 11 July 2025) and conformed to ARRIVE guidelines.

*Cytocompatibility*

Sterile patches were extracted in complete DMEM (10% fetal bovine serum, 1% penicillin–streptomycin; Thermo Fisher Scientific, Waltham, MA, USA) at an area-to-volume ratio of 3 cm$^2$·mL$^{-1}$ for 24 h at 37 °C with gentle agitation; extracts were filtered through a 0.22 μm membrane prior to use. L929 and HaCaT cells were seeded in 96-well plates (≈1.0–1.5 × $10^4$ cells per well) and allowed to adhere overnight, then exposed to extract equivalents of 25%, 50%, and 100% for 24 h. Fresh medium served as vehicle control and 0.2% Triton X-100 (Sigma-Aldrich, St. Louis, MO, USA) as a positive cytotoxic control. Each

condition included n = 3 biological wells × 2 technical replicates. After exposure, MTT solution (0.5 mg·mL$^{-1}$) was added for 3–4 h, crystals were dissolved in DMSO, and absorbance was read at 570 nm with background correction at 630–690 nm on a plate reader available in the facility.

$$\text{Viability (\%)} = 100 \times \text{Abs}_{570}(\text{sample})/\text{Abs}_{570}(\text{control})$$

and the ISO 10993-5 passing threshold was ≥70% mean viability.

### *Serum Biochemistry*

Terminal blood (or blood under anesthesia where permitted) was collected into serum tubes, allowed to clot for 20–30 min at room temperature, and centrifuged at 2,000 g for 10 min. Serum was analyzed the same day on an automated clinical chemistry analyzer available in our facility (reagent kits from Thermo Fisher Scientific, Waltham, MA, USA, or Roche Diagnostics, Indianapolis, IN, USA, as applicable), or stored at 4 °C for <6 h before testing. Laboratory reference intervals for C57BL/6J were considered during interpretation: AST 35–80 U·L$^{-1}$, ALT 25–60 U·L$^{-1}$, urea 5–9 mmol·L$^{-1}$, and creatinine 25–60 μmol·L$^{-1}$ (ranges may vary by laboratory). Hemolyzed samples were excluded per pre-specified criteria. Statistical analyses followed the main text (normality by Shapiro–Wilk, variance homogeneity by Levene, one-way ANOVA with Tukey post hoc; $\alpha = 0.05$).

### *Data processing and analytical QC rules*

ELISA data were processed with validated spreadsheets using 4PL (1/y$^2$) models for calibration, back-calculation of unknowns, and automatic QC/pass-fail flags according to predefined criteria (calibration $R^2 \geq 0.99$, QC recovery 80–120%, CV ≤15%—≤20% at LLOQ, acceptable parallelism and carryover). ICP-MS runs required passing CCB/CCV (bias ≤ ±10%) and demonstrated drift ≤ 5% across each batch; low-level performance informed LOD/LOQ estimates. Outliers, when present, were handled by pre-specified statistical rules (e.g.,

Grubbs' test, $\alpha$ = 0.05) and are documented where applicable in the Supplementary Results.

**Figure S1.** Patch Content Uniformity (ELISA). Histogram of insulin per patch (n = 24) with mean ± SD and CV%.

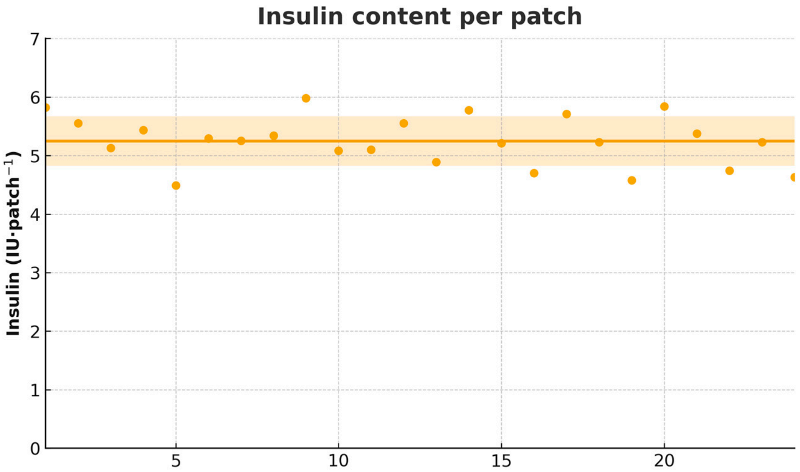

**Figure S2.** Single-needle fracture strength. Distribution across 6 arrays × 20 needles; per-array mean ± SD.

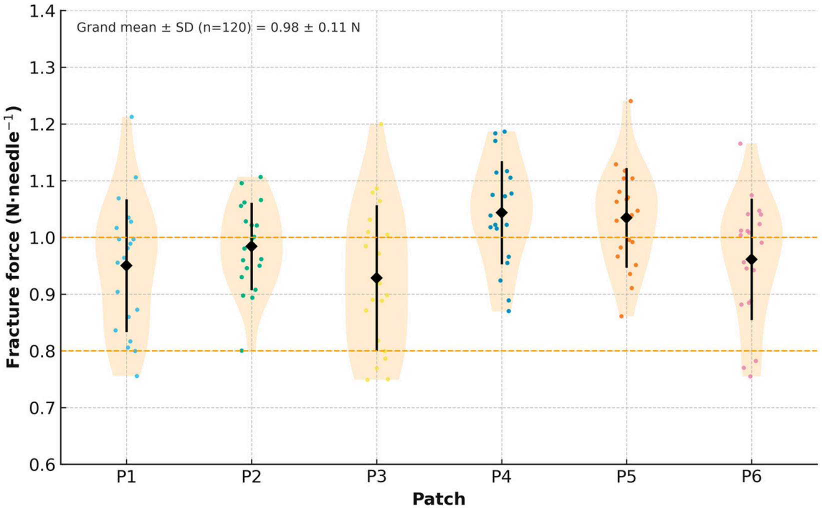


**Table S1.** ELISA Validation — Patch Extract Matrices

4PL calibration achieved $R^2 = 0.9993$ for the run. Acceptability for QCs, spike–recovery, and parallelism were met.

| Std Level | Nominal (ng·mL⁻¹) | Back-Calc (ng·mL⁻¹) | Bias (%) | Accept? | | |
|---|---|---|---|---|---|---|
| **Std 1** | 0.50 | 0.49 | -2.0 | Yes | | |
| **Std 2** | 1.00 | 1.02 | 2.0 | Yes | | |
| **Std 3** | 2.50 | 2.48 | -0.8 | Yes | | |
| **Std 4** | 5.00 | 5.10 | 2.0 | Yes | | |
| **Std 5** | 10.00 | 9.80 | -2.0 | Yes | | |
| **QC Level** | Nominal (ng·mL⁻¹) | Mean (ng·mL⁻¹) | SD | CV (%) | Accept? | |
| **Low** | 0.80 | 0.83 | 0.05 | 6.0 | Yes | |
| **Mid** | 3.00 | 2.94 | 0.12 | 4.1 | Yes | |
| **High** | 8.00 | 8.16 | 0.28 | 3.4 | Yes | |
| **Dilution** | Spike (ng·mL⁻¹) | Measured (ng·mL⁻¹) | Recovery (%) | n | Mean Recovery (%) | Accept? |
| **1:10** | 0.80 | 0.82 | 102.5 | 3 | 102.5 | Yes |
| **1:10** | 3.00 | 2.94 | 98.0 | 3 | 98.0 | Yes |
| **1:10** | 8.00 | 8.24 | 103.0 | 3 | 103.0 | Yes |
| **1:20** | 0.80 | 0.78 | 97.5 | 3 | 97.5 | Yes |
| **1:20** | 3.00 | 3.06 | 102.0 | 3 | 102.0 | Yes |
| **1:20** | 8.00 | 7.76 | 97.0 | 3 | 97.0 | Yes |
| **Dilution** | Expected (ng·mL⁻¹) | Measured (ng·mL⁻¹) | Back-Calc (ng·mL⁻¹) | Bias (%) | Accept? | |
| **1:5** | 6.00 | 5.70 | 5.70 | -5.0 | Yes | |

| | | | | | |
|---|---|---|---|---|---|
| **1:10** | 3.00 | 2.94 | 2.94 | -2.0 | Yes |
| **1:20** | 1.50 | 1.49 | 1.49 | -0.7 | Yes |
| **1:40** | 0.75 | 0.76 | 0.76 | 1.3 | Yes |
| **1:80** | 0.38 | 0.40 | 0.40 | 5.3 | Yes |

Estimated LOD = 0.10 ng·mL$^{-1}$; LOQ = 0.30 ng·mL$^{-1}$. Carryover after ULOQ was 12% of the LLOQ response (acceptable).

4PL parameters. For a representative run, the 4PL fit yielded A = 0.052 (OD), B = 1.12, C = 2.84 ng·mL$^{-1}$, D = 2.01 (OD), $R^2$ = 0.999; LLOQ = 0.30 ng·mL$^{-1}$ (bias within ±20%, CV ≤ 20%), ULOQ = 10 ng·mL$^{-1}$.

Plate layout. Standards and QCs were run in duplicate; samples in duplicate; corner wells were avoided to minimize edge effects. QC pass criteria: 2/3 within 80–120% with CV ≤ 15%.

Matrix factor. Parallelism slopes (matrix vs. diluent) within 0.85–1.15 confirmed minimal matrix effects after ≥1:10 dilution.

**Table S2.** ELISA Validation — Mouse Plasma (PK)

4PL calibration achieved $R^2$ = 0.9991. Selectivity versus endogenous mouse insulin was confirmed by negligible signal in blank plasma (mean blank 0.03 ng·mL$^{-1}$ eq., < LLOQ).

| **Std Level** | **Nominal (ng·mL$^{-1}$)** | **Back-Calc (ng·mL$^{-1}$)** | **Bias (%)** | **Accept?** |
|---|---|---|---|---|
| **Std 1** | 0.50 | 0.51 | 2.0 | Yes |
| **Std 2** | 1.00 | 0.98 | -2.0 | Yes |
| **Std 3** | 2.50 | 2.55 | 2.0 | Yes |
| **Std 4** | 5.00 | 4.90 | -2.0 | Yes |
| **Std 5** | 10.00 | 10.20 | 2.0 | Yes |

| **QC Level** | Nominal (ng·mL$^{-1}$) | Mean (ng·mL$^{-1}$) | SD | CV (%) | Accept? |
|---|---|---|---|---|---|
| **Low** | 0.80 | 0.84 | 0.07 | 8.3 | Yes |
| **Mid** | 3.00 | 3.06 | 0.12 | 3.9 | Yes |
| **High** | 8.00 | 7.76 | 0.32 | 4.1 | Yes |

| **Dilution** | Spike (ng·mL$^{-1}$) | Measured (ng·mL$^{-1}$) | Recovery (%) | n | Mean Recovery (%) | Accept? |
|---|---|---|---|---|---|---|
| **1:5** | 0.80 | 0.76 | 95.0 | 3 | 95.0 | Yes |
| **1:10** | 3.00 | 2.91 | 97.0 | 3 | 97.0 | Yes |
| **1:20** | 8.00 | 8.16 | 102.0 | 3 | 102.0 | Yes |

Stability: bench-top (6 h) 98–103%; 3× freeze–thaw 94–102%; −80 °C (14 d) 95–104% — all within acceptance criteria.

Selectivity & cross-reactivity. Blank mouse plasma produced signals < LLOQ. According to the manufacturer, the assay shows < 0.1% cross-reactivity with mouse insulin; spike recovery 95–102% across 1:5–1:20 dilutions supported target selectivity.

**Table S3.** ICP–MS Validation — Boron

External calibration yielded $R^2$ = 0.9996 for 0–100 µg·L⁻¹. Carryover after the top standard was 4% of the LLOQ response. Instrumental drift over the batch was 2.6%. Estimated LOD = 0.20 µg·L⁻¹; LOQ = 0.60 µg·L⁻¹.

| **Cal Level (µg·L⁻¹)** | **Measured (µg·L⁻¹)** | **Bias (%)** | **n** | **$R^2$ (run)** | **Accept?** |
|---|---|---|---|---|---|
| **0** | 0.02 | | | | — |
| **5** | 5.10 | 2.0 | 1 | 0.9996 | Yes |
| **10** | 10.10 | 1.0 | 1 | | Yes |
| **50** | 49.20 | -1.6 | 1 | | Yes |
| **100** | 99.00 | -1.0 | 1 | | Yes |
| **QC Level** | Nominal (µg·L⁻¹) | Measured (µg·L⁻¹) | Recovery (%) | CV (%) | Accept? |
| **Low** | 3.0 | 2.9 | 98.0 | 3.1 | Yes |
| **Mid** | 30.0 | 30.9 | 103.0 | 2.8 | Yes |
| **High** | 80.0 | 78.4 | 98.0 | 2.5 | Yes |

Notes: PBS-matched QCs controlled ion suppression; blank subtracted; acceptance 80–120% recovery, CV ≤ 15%.

ICP-MS settings. Isotope $^{11}B$ monitored in no-gas mode; internal standard $^{115}In$ 10 µg·L⁻¹ online. Samples were acidified to 1% $HNO_3$ immediately after collection. Rinse protocol: 2% $HNO_3$ + 0.05% HF-free surfactant for 30 s between injections to mitigate boron memory. CCB/CCV were run every 10 samples (bias ≤ ±10%). Measured boron was converted to µg·patch⁻¹·day⁻¹ using leachate volume and patch count.

**Table S4.** STZ Diabetes Induction Outcomes per Animal

| **Mouse_ID** | **BodyWeight_pre (g)** | **BodyWeight_post (g)** | **FPG_pre (mg·dL⁻¹)** | **FPG_post (mg·dL⁻¹)** | **Induction_success** |
|---|---|---|---|---|---|
| **M01** | 23.589 | 20.592 | 102.836 | 444.418 | TRUE |
| **M02** | 24.745 | 23.257 | 105.169 | 466.803 | TRUE |
| **M03** | 27.563 | 27.753 | 102.418 | 222.127 | FALSE |
| **M04** | 19.423 | 18.925 | 95.239 | 385.639 | TRUE |
| **M05** | 21.171 | 21.263 | 98.526 | 385.519 | TRUE |
| **M06** | 21.391 | 20.077 | 102.878 | 380.489 | TRUE |
| **M07** | 20.969 | 19.766 | 106.090 | 355.134 | TRUE |
| **M08** | 20.560 | 19.635 | 112.257 | 316.664 | TRUE |
| **M09** | 21.961 | 19.052 | 117.970 | 389.263 | TRUE |
| **M10** | 20.310 | 18.571 | 101.468 | 349.656 | TRUE |
| **M11** | 23.103 | 21.222 | 99.526 | 388.956 | TRUE |
| **M12** | 22.593 | 22.038 | 110.252 | 348.474 | TRUE |
| **M13** | 23.366 | 22.865 | 112.330 | 334.416 | TRUE |
| **M14** | 23.414 | 22.084 | 99.790 | 183.061 | FALSE |
| **M15** | 23.585 | 22.147 | 114.485 | 391.942 | TRUE |

| | | | | | |
|---|---|---|---|---|---|
| **M16** | 21.828 | 22.013 | 116.755 | 411.647 | TRUE |

Summary: 14/16 success (87.5%). Successful FPG_post mean ± SD = 382.1 ± 39.3 mg·dL$^{-1}$. Body weight change (pre–post) in successful mice = 1.23 ± 0.93 g.

**Table S5.** Cytocompatibility (ISO 10993-5)

| CellLine | Extract_% | Mean Viability (%) | SD (%) | Meets ISO ≥70%? |
|---|---|---|---|---|
| **HaCaT** | 0 | 99.23 | 3.45 | Yes |
| **HaCaT** | 25 | 104.57 | 2.21 | Yes |
| **HaCaT** | 50 | 98.33 | 2.18 | Yes |
| **HaCaT** | 100 | 94.16 | 1.42 | Yes |
| **L929** | 0 | 100.12 | 1.61 | Yes |
| **L929** | 25 | 97.02 | 5.50 | Yes |
| **L929** | 50 | 101.98 | 2.76 | Yes |
| **L929** | 100 | 97.46 | 0.82 | Yes |

**Table S6.** Serum Biochemistry

| Group | AST ($U \cdot L^{-1}$) | ALT ($U \cdot L^{-1}$) | Urea ($mmol \cdot L^{-1}$) | Creatinine ($\mu mol \cdot L^{-1}$) |
|---|---|---|---|---|
| **PBS** | 45.9 ± 5.2 | 31.2 ± 4.9 | 6.2 ± 0.8 | 37.7 ± 3.0 |
| **PDB-MN** | 47.6 ± 5.2 | 32.9 ± 4.1 | 7.0 ± 0.4 | 37.8 ± 7.2 |
| **SC Insulin** | 48.2 ± 6.7 | 32.3 ± 2.8 | 6.7 ± 1.4 | 39.4 ± 2.5 |
| **sI-MN** | 50.8 ± 4.4 | 33.1 ± 2.5 | 6.4 ± 0.9 | 38.0 ± 5.2 |
